# Genome Instability in Multiple Myeloma: Facts and Factors

**DOI:** 10.3390/cancers13235949

**Published:** 2021-11-26

**Authors:** Anna Y. Aksenova, Anna S. Zhuk, Artem G. Lada, Irina V. Zotova, Elena I. Stepchenkova, Ivan I. Kostroma, Sergey V. Gritsaev, Youri I. Pavlov

**Affiliations:** 1 Laboratory of Amyloid Biology, St. Petersburg State University, 199034 St. Petersburg, Russia; 2 International Laboratory “Computer Technologies”, ITMO University, 197101 St. Petersburg, Russia; ania.zhuk@gmail.com; 3 Department of Microbiology and Molecular Genetics, University of California, Davis, CA 95616, USA; alada@ucdavis.edu; 4 Department of Genetics and Biotechnology, St. Petersburg State University, 199034 St. Petersburg, Russia; info@grayhawk.spb.ru (I.V.Z.); stepchenkova@gmail.com (E.I.S.); 5 Vavilov Institute of General Genetics, St. Petersburg Branch, Russian Academy of Sciences, 199034 St. Petersburg, Russia; 6 Russian Research Institute of Hematology and Transfusiology, 191024 St. Petersburg, Russia; obex@rambler.ru (I.I.K.); gritsaevsv@mail.ru (S.V.G.); 7 Eppley Institute for Research in Cancer, Fred and Pamela Buffett Cancer Center, University of Nebraska Medical Center, Omaha, NE 68198, USA; 8 Departments of Biochemistry and Molecular Biology, Microbiology and Pathology, Genetics Cell Biology and Anatomy, University of Nebraska Medical Center, Omaha, NE 68198, USA

**Keywords:** multiple myeloma, genome instability, translocations, chromothripsis, *kataegis*, editing deaminases, DNA repair

## Abstract

**Simple Summary:**

Multiple myeloma is an incurable blood cancer caused by the malignant transformation of immunoglobulin-producing plasma cells. The mechanisms leading to the origin of cancerous cells and the evolution of myeloma disease are not understood. The development of myeloma is accompanied by genetic changes affecting various cellular pathways. This review describes current progress in understanding the etiology of the disease that might stimulate the development of new therapies.

**Abstract:**

Multiple myeloma (MM) is a malignant neoplasm of terminally differentiated immunoglobulin-producing B lymphocytes called plasma cells. MM is the second most common hematologic malignancy, and it poses a heavy economic and social burden because it remains incurable and confers a profound disability to patients. Despite current progress in MM treatment, the disease invariably recurs, even after the transplantation of autologous hematopoietic stem cells (ASCT). Biological processes leading to a pathological myeloma clone and the mechanisms of further evolution of the disease are far from complete understanding. Genetically, MM is a complex disease that demonstrates a high level of heterogeneity. Myeloma genomes carry numerous genetic changes, including structural genome variations and chromosomal gains and losses, and these changes occur in combinations with point mutations affecting various cellular pathways, including genome maintenance. MM genome instability in its extreme is manifested in mutation *kataegis* and complex genomic rearrangements: chromothripsis, templated insertions, and chromoplexy. Chemotherapeutic agents used to treat MM add another level of complexity because many of them exacerbate genome instability. Genome abnormalities are driver events and deciphering their mechanisms will help understand the causes of MM and play a pivotal role in developing new therapies.

## 1. Clinical Manifestation of Multiple Myeloma and Recent Research Approaches

### 1.1. Clinical Characteristics of Pre-MM and MM: Heterogeneity and Clonal Evolution of Cancer Cells

**Figure 1:**
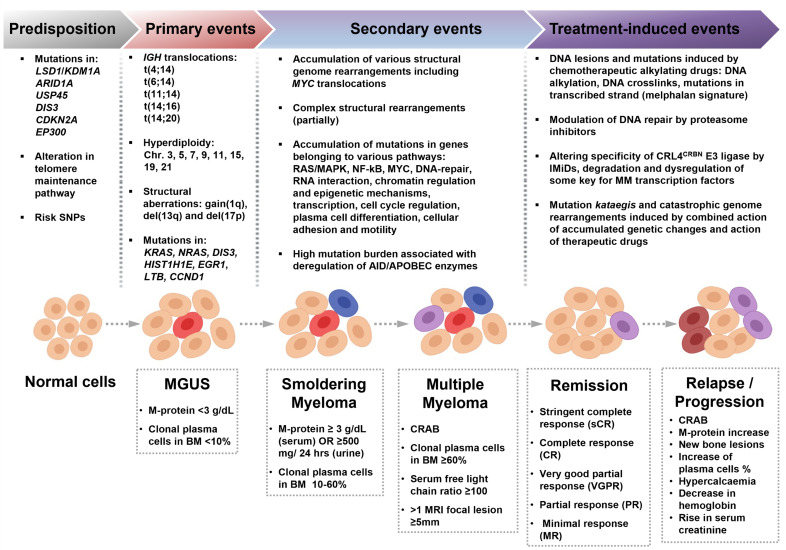
Stages of MM development and their characteristics. MM is almost always preceded by a precancerous condition termed monoclonal gammopathy of undetermined significance, MGUS [5,6,7]. Smoldering multiple myeloma (SMM) is an intermediate stage between MGUS and MM [12,13]. MGUS is diagnosed when serum monoclonal protein (M-protein) is detected, but levels are lower than in MM; a 2–3-fold elevation of the number of clonal bone marrow plasma cells is usually found in MGUS compared to healthy individuals. In MGUS, end-organ damage, CRAB, attributed to the plasma cell proliferative disorder, is absent [1]. SMM is diagnosed when serum or urinary monoclonal protein rises and/or the number of clonal bone marrow plasma cells increases, but there is no MDE or amyloidosis [1]. There are germline risk alleles in several genes associated with familiar cases of MM and MGUS; also, numerous SNPs found in GWAS are associated with MM development risk. Telomere status is another factor that can affect MM development. Primary events can be found as early as MGUS and are represented by structural genome changes, gain of chromosomes, and mutations. The primary structural genomic changes are nearly equally represented by the two major groups: the *IGH* translocations and trisomies of several odd chromosomes (referred to as hyperdiploidy). Few other structural aberrations found as early as the MGUS stage are listed. While the disease progresses, more genomic changes accumulate. The events that are characteristic of SMM and MM, but not MGUS, are classified as secondary. They are represented by various structural genome changes including *MYC* translocations, accumulation of complex genome rearrangements (partially, since some of them can be seen at MGUS stage), mutations in various pathways, and AID/APOBEC-induced mutation burden. The endpoint of the scheme illustrates treatment-induced events. Some of them can modulate DNA repair and impact genome stability and mutation accumulation. Abbreviations: IMiDs—Immunomodulatory drugs; CRAB—an acronym for Calcium (elevated), Renal failure, Anemia, and Bone lesions, the most common symptoms of MM; MDE—myeloma-defining events (see text for more details); MRI—magnetic resonance imaging; BM—bone marrow; AID—activation-induced cytosine deaminase essential for class switch recombination (CSR) and somatic hypermutation (SHM); APOBEC—a family of cytosine deaminases that play an essential role in mutagenesis in cancer cells; GWAS—Genome-Wide Association Studies.

MM is characterized by aberrant expansion of terminally differentiated monoclonal plasma cells resulting in symptoms described by the acronym “CRAB”: hypercalcemia, renal failure, anemia, and bone lesions. In addition to that, diagnostic criteria include three biomarkers of malignancy: the presence of excessive clonal bone marrow plasma cells, elevated serum free light chain ratio (ratio of κ to λ free light chains), or focal bone lesions [1,2,3] (Figure 1). These symptoms and biomarkers are called myeloma-defining events (MDE) [1]. At least one MDE in addition to a biopsy-proven plasmacytoma or ≥10% of plasma cells in bone marrow is required for the MM diagnosis [1]. Development of MM is a multi-stage process beginning from a premalignant stage termed monoclonal gammopathy of undetermined significance (MGUS) [4,5,6]. MGUS can be accidentally found years to decades before the actual diagnosis of MM. It could be present in ~3% of the normal human population over 50 years old [7]. MGUS does not necessarily develop into MM; further progression of MGUS into the active MM has linear risk and occurs with a rate of ~1% per year [1,8,9,10,11].

An additional, more advanced stage is observed in some patients, which is referred to as smoldering multiple myeloma (SMM) [1,11,12,13,14]. MGUS and SMM are usually asymptomatic stages characterized by different levels of M-protein production and different ratios of clonal plasma cells in bone marrow (see Figure 1). SMM may represent asymptomatic MM rather than being an MM precursor [15]. Patients with SMM follow a declining logarithmic progression curve to symptomatic MM: 10% risk per year for the first 5 years following diagnosis, 3% risk per year for the following 5 years, and a subsequent 1% risk per year [1,8,13]. Both MGUS and SMM have specific diagnostic criteria (Figure 1). MM and its precursors can produce different types of monoclonal proteins. For instance, the production of different isotypes of the immunoglobulin heavy chain has been described in MGUS [7]. The two clinically significant entities are IgM MGUS and non-IgM MGUS demonstrating different chances of progression into the MM. While the non-IgM MGUS is associated with a risk of progression to MM, IgM MGUS most frequently progresses into non-Hodgkin lymphoma and its subtype Waldenström macroglobulinemia [10,16,17]. In addition, no immunoglobulin heavy chain production can be seen in some cases in patients with abnormal serum free light chain ratio, which is attributed to the light chain MGUS and the light chain MM [6,18]. An uncommon subtype of MM is non-secretory myeloma, found in 2–3% of all MM cases [1,18,19].

The biological processes leading to the appearance of a pathological myeloma clone and the mechanisms of the disease evolution are not yet deciphered. Factors responsible for the emergence of MM include a combination of genetic predisposition, alterations in genomes of the lymphoid cells (such as *IGH* translocations or hyperdiploidy), and a variety of secondary changes, which include accumulation of mutations, chromosomal rearrangements, and complex genetic events (Figure 1). Apparent inefficiency of the initial treatment, fast recurrences, resistance to the earlier prescribed drugs, and clonal behavior of the disease implies that MM represents a heterogeneous entity. Multiple studies suggest that newly diagnosed MM represents an aggregate of the main pathological clone with several subclones that acquire proliferative priority when the main clone is suppressed during specific therapy [20,21,22,23,24,25,26,27,28,29,30,31,32,33,34].

The classic clonal evolution implies the sequential acquisition of mutations with a concomitant sequential selection of successive subclones, their expansion, and mutual interference [35,36]. Each tumor cell can carry many genetic abnormalities, including mutations that provide a selective growth advantage: “driver” mutations, selectively neutral “passenger” mutations, and deleterious mutations affecting fitness [37]. In addition to that, there are “mutator” mutations that increase the rate of genetic changes. The dynamics of tumor evolution is a function of mutation rate elevation and clonal expansion that relies on “driver” mutations. Natural selection provides “selective sweeps” when one or several clones grow to dominate the neoplasm [36]. These dominant clones accumulate new genetic changes in addition to the mutational landscape of the original tumor as the evolution proceeds. The complexity of this process is augmented by epigenetic changes that, similar to changes in the DNA, can confer either growth advantage or be selectively neutral or deleterious. Epigenetic changes can also affect mutation rates. On top of this complexity is the impact of therapeutic drugs, which can modify DNA, affect DNA repair processes, and modulate the growth advantage of the cancer cells [38,39,40].

The clonal development of the tumor may follow the linear model if driver changes provide a strong selective advantage that outcompetes all previous clones, be branched if several clones expand simultaneously, or neutral if there is no selective advantage between multiple clones’ appearance and co-existence. Alternatively, most essential changes may occur simultaneously or near-simultaneously early in tumor development, establishing several dominant clones that grow stably, a characteristic of punctuated evolution [36,41]. It is generally recognized that the MM development follows the rules of branched evolution [27,42,43]. However, patterns of clones’ phylogeny consistent with linear, neutral, and punctuated models of evolution have also been described during MM development and progression [22,27,29,44,45]. Moreover, recent advances in studies of MM and its precursors have centered the punctuated evolution model as characterizing best the early stages of the MM development [20,46,47,48,49]. For instance, significant heterogeneity is present before the disease manifestation at the MGUS and SMM stages [20,23,30,47,48,50,51,52], and further progression from MGUS/SMM to MM is characterized by clonal stability in some tumors [20,46,47,48]. Importantly, clonal evolution in MM seems to be closely interrelated with treatment strategies [22,23,24,25,26,28,34,45,53,54]. As the disease proceeds, the variability of the clones increases, which creates the ground for further clonal diversification and evolution [23,45,47,51,52,53,55,56,57,58,59,60,61,62,63]. One or several such clones will ultimately lead to the recurrence of myeloma. It is possible that the change from the main clone to subclones during MM treatment causes a change in the clinical and hematological phenotype of MM and, thus, explains the inefficiency of the previously conducted therapy. Noteworthy, patients with MM or its precursors have an increased risk of developing secondary primary malignancies (SPMs), such as myelodysplastic syndrome (MDS), acute myeloid leukemia (AML), and others [64,65,66,67]. The origin of these tumors is likely related to the genotoxic action of some therapeutic agents [65,68,69,70,71,72,73,74], although an excess risk for hematopoietic neoplasms other than MM in MGUS patients supports an idea that endogenous factors play an essential role in SPMs’ development [9,75,76,77]. Therefore, it is possible that the genetic landscape of hematopoietic stem cells in MM patients may predispose them to different malignant programs that can unfold spontaneously or as a result of therapeutic intervention.

Recent developments and advances of next-generation sequencing technologies (NGS) help to produce a large amount of genomic data that are invaluable for MM diagnosis, choices of possible treatment, assessment of drug response, and understanding of disease evolution [78,79,80]. During the last decade, dozens of large-scale NGS studies have been undertaken to find peculiarities of the MM genomes and pinpoint genetic drivers of MM (Table S1) [28,29,61,81,82,83]. Whole-exome (WES) or whole-genome (WGS) sequencing of MM genomes allows for detecting point mutations, small insertions or deletions, and structural variations. Many studies combine their own NGS data with the publicly available datasets from Multiple Myeloma Research Foundation (MMRF) CoMMpass Study (Clinicaltrials.gov identification number: NCT01454297) [84]. Sequencing data for MM samples are deposited in the European Genome Archive or at the Genotype and Phenotype database (dbGaP). The Multiple Myeloma Genome Project (MGP) attempts to assemble and analyze NGS data for MM, improve clinical testing, and define treatment strategies for MM patients [85,86]. MGP provides the repository of WES, WGS, and RNA-Seq data for patients with MM derived from different sources, such as the Multiple Myeloma Research Foundation, the Myeloma XI trial, and others [85].

### 1.2. Current Treatment Algorithms of MM: Mechanisms of Action of Anti-MM Agents

**Figure 2:**
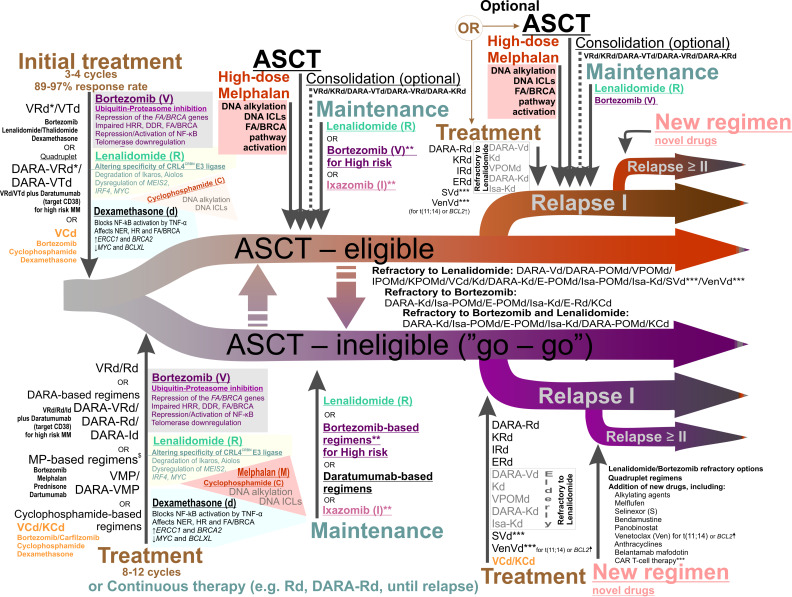
MM treatment outlines. The schemes align with the latest recommendations and studies and include available options for MM treatment as well as standards of care [87,88,90,91,103]. Action of some drugs on DNA integrity or pathways affecting DNA metabolism/repair is shown on the scheme. All patients can be subdivided into three categories based on frailty: “go-go”, “slow-go”, and “no-go” [111]. “Slow-go” and “no-go” categories are usually treated using the reduced intensity regimens or dose-adjusted regimens and are not shown on the scheme. Treatment for the “no-go” category excludes chemotherapeutic drugs and includes agents with low toxicity, mostly for palliative care [111]. The “go-go” category and to some extent the “slow-go” category are shown on the scheme. VRd * can be used as induction (initial) therapy for newly diagnosed and transplant-eligible patients with MM and is associated with a progression-free survival longer than 4 years and overall survival at 4 years higher than 80% [87,103]. The alternative scheme is VTd, which is slightly less efficient [103]. The addition of the monoclonal antibody daratumumab targeting CD38 to the standard VRd or VTd regimen (Quadruplet regimen, Dara-VRd *, or Dara-VTd) is recommended for high-risk patients [87]. DARA-Rd is also an important alternative to VRd in newly diagnosed MM [112]. Besides that, VCd can be used as an induction scheme [88]. High-dose melphalan and ASCT give a good response and provide the longest remission [87,102]. Single-agent lenalidomide, administered continuously until disease progression is considered the standard of care for maintenance, and bortezomib can be used for high-risk patients. In addition, ixazomib can be considered in place of bortezomib [87,103,113]. If the disease relapses, another ASCT can be tried for eligible patients [100,101,102,114,115]; new treatment regimens are usually considered, including quadruplet schemes and new drugs [87]. The alkylating agent cyclophosphamide can be used instead of immunomodulatory agents as treatment for refractory patients and in patients with renal dysfunction in combination with bortezomib and dexamethasone; this setting can also be used for induction therapy in ASCT-eligible patients [87,116,117,118,119,120,121,122,123,124,125]. Melphalan and prednisone-based therapy$ can be used for ASCT-ineligible patients and often is represented by VMP or DARA-VMP schemes [88]. Continuous therapy or initial therapy with maintenance can be used in a non-transplant setting [113]. A multi-regimen, including bortezomib, an immunomodulatory drug, dexamethasone, cytotoxic cisplatin, doxorubicin, cyclophosphamide, and etoposide, may be used for plasma cell leukemia or extramedullary disease [87]. Schemes that can be used for refractory to lenalidomide and/or bortezomib patients are also given in the center of the figure. Abbreviations: V—bortezomib, DARA—daratumumab, R—lenalidomide, T—thalidomide, POM —pomalidomide, d—dexamethasone; K—carfilzomib, I—ixazomib, E—elotuzumab, Isa—isatuximab, C—cyclophosphamide, P—prednisone; S—selinexor, Ven—venetoclax, ICL—intermolecular crosslinks in the DNA, FA/BRCA—pathway responsible for the repair of intermolecular crosslinks in the DNA, HR—homologous recombination, HRR—homologous recombination repair, DDR—DNA damage response, NER—nucleotide excision repair. *—Lenalidomide-containing regimens (e.g., VRd and Dara-VRd) are not yet approved by European Medicines Agency (EMA, EU) as induction for ASCT-eligible patients, although they offer good risk–benefit profiles and are widely used in the USA [87,88]. **—Bortezomib and ixazomib have not yet been approved by the EMA for maintenance after ASCT [88,113]. ***—Awaiting EMA approval. $—melphalan-containing regimens in this setting are not recommended in the USA due to concerns about SPMs and stem cells damage.

MM patient management strategy differs significantly depending on age, comorbidities, cytogenetic parameters, disease stage, risk stratification, and other factors [87,88,89,90,91] (Figure 2). For the treatment of primary patients under 70 years of age without serious comorbidities, high-dose chemotherapy (HDCT) with autologous hematopoietic stem cell transplantation (ASCT) is included in the treatment program (Figure 2). Several recent studies show that ASCT can be a safe choice for patients older than 70 years [92,93,94,95,96]. Despite significant toxicity, ASCT remains the standard of care and is associated with prolonged progression-free survival and better overall survival [87,88,90,97,98,99,100,101,102,103,104,105,106,107,108,109]. ASCT can be delayed in standard-risk patients if they respond to the induction treatment [87,110]. HDCT with concomitant ASCT is not recommended for patients with comorbidities and for frail patients [87,88]. Examples of treating schemes for ASCT-ineligible patients are shown in Figure 2.

The therapeutic arsenal for MM treatment includes DNA damaging agents (melphalan, cyclophosphamide, etc.), immunomodulatory agents (IMiDs, e.g., thalidomide, lenalidomide, pomalidomide), proteasome inhibitors (PIs, e.g., bortezomib, carfilzomib, ixazomib), monoclonal antibodies (daratumumab, isatuximab, elotuzumab), and corticosteroids [1,87,91]. In addition to that, several drugs can be used in combination with other therapies as they are not considered efficient alone in most cases. These include inhibitors of histone deacetylase 6 (panobinostat), an inhibitor of Exportin-1 (selinexor, considered efficient in combination with dexamethazone), the DNA intercalating drugs anthracyclines (doxorubicin), and the BCL2 inhibitor venetoclax, which does not yet have approval for treatment of MM but appears to be efficient against MM with t(11;14) rearrangement [87,126,127]. Besides that, new agents and therapies have been approved or are about to be approved for the treatment of MM. They include an immunoconjugate drug targeting B-cell maturation antigen, BCMA (Belantamab Mafodotin), an inhibitor of kinesin spindle protein, KSP (Filanesib), chimeric antigen receptor (CAR) T-cell therapy (e.g., bb2121 therapy that targets BCMA), and bispecific antibodies [128,129,130,131,132,133,134].

Many agents used in MM treatment are genotoxic and can further elevate the genetic variability of MM, which can be harmful and potentiate disease progression and relapse [25,135]. The consequences of using one or another agent for the genome differ depending on the preexisting genomic alterations and mutations. Drugs within the chemotherapeutic arsenal with potential genotoxicity include alkylating DNA agents (melphalan, cyclophosphamide, bendamustine, busulfan), intercalating DNA agents (doxorubicin), microtubule-depolymerizing drugs (vincristine), agents inactivating topoisomerase II (etoposide, doxorubucin), and crosslinking agents (cisplatin). Melphalan is a phenylalanine-substituted derivative of nitrogen mustard that alkylates adenine and guanine in the DNA. It has two highly reactive chloroethylamine groups and thus induces intra- or intermolecular crosslinks in the DNA and between DNA and proteins [136,137,138]. The high-dose melphalan is a standard regimen before ASCT [87,103,136,139,140]. Cyclophosphamide also belongs to a family of mustard-alkylating agents and induces alkylation and crosslinking of the DNA [141,142,143]. At low doses, cyclophosphamide demonstrates immunomodulatory activity [144]. Notably, a significant increase in mutation burden and specific mutational signature have been reported in MM patients exposed to high-dose melphalan [54,59,63,145,146]. Melphalan (and to a lesser extent cyclophosphamide) is also associated with an increased risk of SPMs in MM patients [64,68,69,71,72,147,148,149,150]. The line of chemotherapeutic drugs has been consistently elaborated upon with novel therapeutic agents in recent years. One of them, a peptide-conjugate alkylator drug, melphalan flufenamide (melflufen), is currently in clinical trials [151,152,153]. Alkylating and crosslinking agents induce modifications to DNA that are a subject of DNA repair to allow for DNA replication and transcription [154]. The vulnerability of cancer cells to these drugs depends on the competing processes of cell cycle progression and repair. Base excision repair (BER), nucleotide excision repair (NER), and mismatch repair (MMR) remove monoadducts in the DNA, intra-strand crosslinks, and other lesions that affect only one strand of the two strands of the duplex DNA [155]. The DNA double-strand breaks (DSBs) interrupt the continuity of the DNA molecule and are repaired via two major repair routes: homologous recombination repair (HRR) and nonhomologous end-joining (NHEJ) [155]. The first one uses the homologous sequence as an instruction to recover the lost information, and the second one just joins the two ends together and seals the break. Despite their provisory division into error-free and error-prone pathways, correspondingly, both HRR and NHEJ can lead to point mutations and chromosome rearrangements. Inter-strand crosslinks constitute a physical barrier to the progression of both RNA and DNA polymerases since they impede the unwinding of the double helix. This type of lesion requires the action of multiple players belonging to the BER, NER, MMR, and HRR pathways and are grouped into a specialized FA/BRCA pathway [156,157]. FA/BRCA was named after Fanconi anemia (FA), a genetic disorder leading to developmental abnormalities and predisposition to cancer development [158]. Mutations in the FA/BRCA pathway genes make cells hypersensitive to agents inducing DNA crosslinks [156,159]. Upregulation of the FA/BRCA pathway contributes to acquired resistance to melphalan in MM cell lines [160]. Human myeloma cell lines frequently acquire mutations in the genes of the FA/BRCA pathway [161]. Selinexor, an inhibitor of the nuclear Exportin 1 (XPO1), decreases the expression of the FA/BRCA and NF-kB pathway genes, reduces melphalan-induced monoubiquitination of FANCD2, and overcomes the resistance of the MM cell lines to melphalan [162]. In addition to that, several other key factors of HR and NHEJ are found upregulated in MM cell lines, which can have implications to MM resistance to DNA-damaging drugs [163,164,165,166]. Bendamustine is a derivative of mechlorethamine that acts both as an alkylating agent and purine analog [167,168]. It has anti-MM activity and can overcome resistance to melphalan in MM cell lines [169]. It is most often used for the treatment of relapsed or refractory-to-other-regimens MM and patients with renal impairment not eligible for ASCT but it can be used as a conditioning therapy before ASCT when combined with melphalan [140,170,171,172,173,174,175,176]. Bendamustine efficiently activates the DNA damage response and has a synergistic effect with alkylating agents and pyrimidine analogs in killing MM cell lines [168].

**Figure 3:**
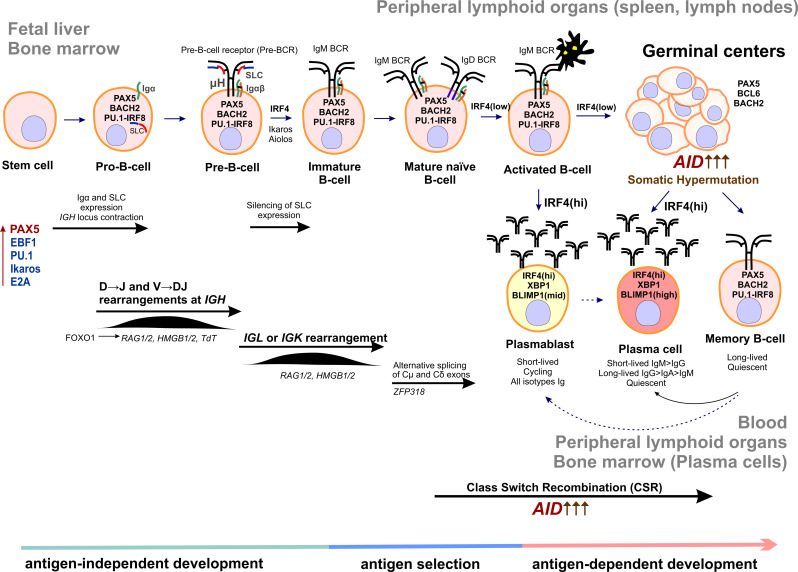
A simplified scheme of B-cell development based on studies in humans and mice. The scheme illustrates the principal stages of B-cell development (predominantly of the B2 lineage) based on studies in mice and humans. Commitment to the B-cell lineage requires the action of several transcription factors (some of them are illustrated in the figure). *PAX5*, *BACH2*, *PU.1*, and *IRF8* are expressed throughout B-cell development and are silenced in antibody-secreting cells [212,213,214]. Surrogate light chains, SLC (VpreB, and λ14.1), and Igα production are the hallmarks of the pro-B-cell stage. V(D)J rearrangement results in the synthesis of the immunoglobulin heavy chain (µH) with Cµ constant domain chain. The µH together with SLC and Igαβ form a pre-BCR receptor, which is required to pass the first essential quality control during B-cell development, after which a rearrangement at the *IGL* or *IGK* loci encoding the λ or κ light chains is initiated. IRF4, along with IRF8, interacts with PU.1 and binds to Ig κ 3′ enhancer and λ enhancers to modulate rearrangement of Ig light chains at the pre-B cell stage [213,215]. This permits the production of the conventional IgM molecules on the cell surface, which corresponds to the “Immature B-cell” stage. The IgM BCR passes rounds of controls to eliminate self-reactive molecules. Dual expression of the IgM BCR and IgD BCR on the cell surface identifies the “Mature B-cell” stage. The mature naïve B-cells migrate to the periphery into the secondary lymphoid organs, where they are subjected to antigen presentation and selection. Upon antigen stimulation, they might form special structures called Germinal Centers (GC), where the antigen-binding sites of the antibodies may be further adaptively altered in the course of SHM, allowing the production of high-affinity antibodies. Starting from this stage, the B-cell may become either a memory cell or the antibody-secreting plasma cell [212,216]. Another type of antibody-secreting cell is plasmablast, plasmablasts are cycling cells that are produced early in the immune response. *IRF4*, along with *BLIMP1(PRDM1)* and *XBP1*, are expressed in antibody-secreting plasma cells and play a role in plasma cell commitment. *IRF4*, *BLIMP1*, and *XBP1* are often found mutated or dysregulated in MM (see Section 3 and Table S2). *IRF4* is induced by BCR signaling and upregulates *BLIMP1*, which has positive feedback on *IRF4* [212]. BLIMP1 is a master-regulator that represses *PAX5* and *BCL6* programs, induces the expression of *XBP1,* and turns the program toward plasma cell development [217]. IRF4 upregulates *AICDA*, encoding AID, and is essential for CSR. CSR is ceased through the BLIMP1-mediated pathway upon B-cell differentiation into plasmablasts or plasma cells [214,217].

IMiDs are derivatives of the teratogenic drug thalidomide that are approved by the US FDA for the treatment of MM [87,120,177,178,179,180,181,182]. Lenalidomide is the standard-of-care maintenance therapy for patients with standard risk and after ASCT [87,97,183,184,185,186,187,188]. Thalidomide, lenalidomide, and pomalidomide bind to Cereblon (CRBN) [177,189,190,191,192]. CRBN, together with DNA Damage Binding Protein-1 (DDB1), Cullin-4A (Cul4A), and Regulator of Cullins-1 (Roc1), forms a complex called Cullin-RING ligase 4 (CRL4^CRBN^), an E3 ubiquitin ligase; CRBN functions as a substrate receptor [191]. The drugs inhibit the binding of CRL4^CRBN^ to its endogenous substrate, transcription factor MEIS2, which regulates MM cell survival and sensitivity to anti-MM drugs [191,193]. CRBN in the presence of drugs acquires the ability to target two specific B cell transcription factors, Ikaros (IKZF1) and Aiolos (IKZF3), for proteasomal degradation [194,195,196] (Figure 2 and Figure 3). Ikaros and Aiolos regulate the expression of the key plasma cell differentiation transcription factor, IRF4, which plays a central role in the pathogenesis of MM [197,198] (Figure 3). *IRF4* controls B-cell-to-plasma-cell program transition and is essential for Germinal Centers (GC) formation, CSR, and SHM [199]. The *AICDA* gene encoding AID cytosine deaminase essential for CSR and SHM (Figure 3) is among the IRF4 targets [200]. *IRF4* is often overexpressed in MM as a result of mutations, translocations, or other events [201,202,203]. It is of interest that lenalidomide can partially affect the IRF4 level [194,195,197,204]. In addition to that, thalidomide and its derivatives can affect the level of cytokine production since they can inhibit TNF-α and IL-1b production and increase IL-2 and IL-10 production [194,205,206]. Inhibition of NF- κB activation has been reported for IMiDs [207,208]. Lenalidomide can affect the levels of other factors, such as KPNA2 and SALL4 [196,209,210]. It is noteworthy that levels of some of these factors, e.g., KPNA2, can be linked to the overall survival and progression-free survival rates in MM patients [209]. It should be noted that some studies have reported increased risk of SPMs associated with lenalidomide [64,65,74,186,211].

Proteasome inhibitors have the potential to affect multiple aspects of cell homeostasis and metabolism, including DNA damage repair pathways [218,219]. Inhibition of proteasome leads to accumulation of misfolded and unfolded proteins in the endoplasmic reticulum, activates the unfolded protein response, and induces apoptosis; remarkably, the unfolded protein response plays an essential role in antibody-secreting cells [216,220,221,222,223,224,225,226]. A potent reversible PI, bortezomib, has shown more than 60% of positive responses in the initial treatment of MM and ~35% of responses for relapsed MM and is routinely used in schemes for induction therapy and as maintenance therapy for high-risk patients [1,87,88,224,227,228,229,230,231,232,233,234,235,236,237,238]. Two other agents inhibiting proteasome function, carfilzomib and ixazomib, are also used for MM treatment and are superior to bortezomib for the treatment of relapsed or refractory MM [1,88,219,239,240,241,242,243,244]. Such drugs with such a broad spectrum of action have a profound influence on DNA metabolism. Bortezomib affects several DNA repair transactions and can sensitize cancer cells for other DNA-damaging therapeutic drugs. First, bortezomib inhibits the FA/BRCA pathway gene expression and leads to transcriptional repression of *FANCD*2, *BRCA1*, *BRCA*2, and *RAD51* genes [245,246]. Second, it reduces monoubiquitination of FANCD2 and polyubiquitylation of γH2AX in cancer cell lines and inhibits the formation of DNA damage foci (BRCA1 and RAD51 foci) in MM cell lines [246,247,248]. Importantly, PIs can affect the NF-κB transcription activation pathway, which is often found dysregulated in MM [207,249,250,251]. Besides that, bortezomib inhibits transcription of the *MMSET* [252]. *MMSET* (multiple myeloma SET domain) encodes for a histone methyltransferase involved in transcription regulation and DNA repair. It is also an oncogene that is highly expressed in diverse tumor types. In the case of MM, *MMSET* is upregulated as a result of the t(4;14) translocation, and its level correlates with the viability of MM cells (Figure 1, see also Section 5.1) [253,254,255]. MMSET, together with NF-kB, is involved in the *IRF4* transcription regulation, and bortezomib can downregulate the *IRF4* [252]. In addition to the discussed mechanisms, PIs can target telomerase (see Section 6).

Histone deacetylases (HDACs) are involved in many cell processes, from chromatin organization and regulation of transcription to protein stability, trafficking, and degradation. Overexpression of some of HDACs is associated with an adverse prognosis in MM [256]. HDAC inhibitors have been found to inhibit MM cell growth and have demonstrated synergistic activity with bortezomib against MM cell lines [257,258]. Panobinostat is approved for the treatment of relapsed and refractory MM in combination with bortezomib and dexamethasone [259,260]. Panobinostat is a pan-HDAC inhibitor that affects many aspects of cell metabolism [261]. A combination of panobinostat and bortezomib exerts a complementary inhibitory effect on protein degradation systems, including proteasome and aggresome; the latter is inhibited through HDAC6, a master regulator of the cell response to cytotoxic misfolded proteins [256,262]. Panobinostat induces acetylation of histones H3 and H4, activation of caspases 3 and 8, and reduces the levels of the transcription factors IRF4 and MYC [263,264]. *MYC* is a well-known oncogene that is deregulated in many cancers, including MM. As a part of the B-cell development, program *MYC* regulates GC formation and is silenced upon switching to the long-lived plasma cell program [198].

Anti-CD38 antibodies have shown efficacy for the treatment of MM in multiple clinical trials in recent years and are included in many treatment schemes [87,88,265,266,267,268]. CD38 is a multifunctional glycoprotein with a high presence on the surface of plasma cells [269,270]. CD38 functions as a cell receptor, adhesion molecule, and ectoenzyme involved in the modulation of many essential cell processes, including immune cell response and signaling [271]. Daratumumab, the anti-CD38 antibody, is approved for MM treatment as a single agent or in combination with other therapies [87,270,272]. Daratumumab induces complement-dependent cytotoxicity and antibody-dependent, cell-mediated cytotoxicity and phagocytosis [269]. Daratumumab significantly reduced the risk of MM progression or death in various groups of patients obtaining different combinations of therapy [273]. The addition of daratumumab to therapy is recommended for high-risk MM patients with refractory/relapsed MM or carrying genetic abnormalities associated with adverse prognosis (see Section 5 for more details) [87]. Remarkably, CD38 is involved in nicotinamide adenine dinucleotide (NAD+) metabolism. While daratumumab seems not to affect NAD+ levels, extracellular and intracellular NAD+ levels may be sensitive to other anti-CD38 molecules such as isatuximab [274,275]. NAD+ is an essential cofactor of many enzymes involved in the regulation of DNA repair and genome stability, including Sirtuins and poly(ADP-ribose) polymerases. NAD+-dependent Sirtuin deacetylase activity is highly sensitive to NAD+ concentrations [271,276]. It is thought that fluctuations in the intracellular level of NAD+ might contribute to genome stability and response to DNA damage, and NAD+-depleting agents might potentiate the benefits of anti-CD38 agents [271,274,277].

Another monoclonal antibody used to treat MM, elotuzumab, targets the signaling lymphocytic activation molecule F7 (SLAMF7), which is highly expressed in plasma cells [1,278,279,280,281]. In addition to that, soluble SLAMF7 has been detected in MM patients but not in healthy individuals [282]. Elotuzumab is not effective in monoregimen against MM but exerts its action in combination with IMiDs [280,281,283]. It promotes an immune response to MM through facilitating natural killer cell-mediated anti-tumor activity [278,284,285]. It is noteworthy that, similar to many other important factors for MM development, the transcription of *SLAMF7* is regulated by MMSET [286].

Inhibitors of the antiapoptotic BCL2-family proteins, including BCL2, MCL1, and BCLXL, are in clinical trials, and some of them can be considered for MM treatment. Proteins of the BCL2-family are essential factors of genome stability. BCL2 directly interferes with ribonucleotide reductase activity and affects replication fork progression and DNA damage repair; it also inhibits repair of the DSBs by influencing NHEJ and HR [287]. MCL1 is found at sites of DNA damage and regulates the DNA damage response [288]. Depletion of MCL1 impairs DNA DSB repair and replication re-initiation at stalled forks, thus increasing genomic instability and cell sensitivity to ionizing radiation [289]. MM cell lines are dependent on MCL1 and are sensitive to its inhibition [290,291,292]. Venetoclax is a selective inhibitor of BCL2 approved for treating chronic lymphocytic leukemia (CLL), a BCL2-dependent malignancy of differentiated B-cells [293]. Venetoclax inhibits BCL2 but not MCL1 or BCLXL. However, MM patients with t(11;14) translocation have increased expression of *BCL2* and are often sensitive to venetoclax [255,293,294]. It has also been shown that bortezomib can downregulate *MCL1* expression and mitigate resistance to venetoclax in some cases [295].

Dexamethasone and prednisone are corticosteroid anti-inflammatory agents routinely used in different combinations to treat MM [87,88]. Dexamethasone blocks NF-kB pathway activation and affects the expression of many genes, including genes implicated in DNA repair pathways and DNA damage response. For instance, it activates the expression of *ERCC1* and *BRCA2* and downregulates *MYC* and *BCLXL* [296,297]. It should be noted that corticosteroids are included in most schemes for MM treatment and may exert a certain effect on genome stability and mutagenesis, especially when used in combination with other drugs influencing DNA metabolism.

## 2. Predisposition to MM

There is strong evidence for familial clustering of MM and MGUS, which suggests that predisposition to MM is inherited [105,298,299,300,301,302,303,304]. Mutations associated with familial risk of MM development affect the genes that encode for the components of chromatin-remodeling complexes and chromatin-modifying enzymes, DNA repair factors, and RNA processing enzymes as well as cell cycle-regulating factors.

Truncating mutations in the gene *LSD1/KDM1A* encoding for lysine-specific histone demethylase 1A (LSD1), a component of the NuRD, CoREST, and SIN3A epigenetic regulatory complexes, are associated with predisposition to MM and early onset of the disease [253,305,306,307,308,309]. LSD1 erases mono- and dimethyl groups from H3K4 (active chromatin) and H3K9 (repressed chromatin), and its substrate specificity largely depends on its interacting partners, thus allowing LSD1 to function either as a transcriptional repressor or coactivator of transcription [310,311,312]. LSD1 plays a role in the regulation of stem-cell programs, and it is overexpressed in different cancers, including hematological malignancies [313,314]. In cooperation with CoREST, LSD1 controls hematopoietic cell differentiation [306]. Transcriptional repressor BCL6 recruits LSD1 to the intergenic and intronic enhancers of many genes controlling B-cell development and GC formation as well as to Germline Transcription (GLT) promoters at the *IGH* locus [315,316]. LSD1 copurifies with MMSET as a part of the SIN3A/HDAC complex, and this interaction might play a role in transcription repression of a number of oncogenes, including telomerase reverse transcriptase *TERT* and regulator of the p53 tumor suppressor *MDM2* [253,309]. Besides that, LSD1 directly participates in the regulation of p53 activity through its demethylation [317]. STAT3 and RB1 are two other non-histone proteins whose activity is regulated by LSD1 [314]. Interestingly, some data suggest that pomalidomide and lenalidomide can activate LSD1, which in turn activates transcription of the cell cycle regulator p21^WAF−1^ [318].

Pedigree analysis identified that mutations in *ARID1A,* encoding a key subunit of SWI/SNF chromatin-remodeling complex, are associated with susceptibility to MM [319]. Genes encoding components of SWI/SNF complexes are often found altered in different cancers [320]. Curiously, *ARID1A* has the highest mutation rate among the genes encoding for the SWI/SNF subunits altered in cancers that can be caused by conditions provoking microsatellite instability [321,322]. There are two main types of SWI/SNF complexes in human cells, classified by their subunit composition: BAF complexes and PBAF complexes; ARID1A is a component of BAF, the complex that interacts directly with topoisomerase IIa (TOP2A) and contributes to the DNA decatenation [323]. Inactivation of BAF functions might result in mitotic defects, chromosomal instability, and polyploidy that are all primary events in cancer development. In addition to that, ARID1A binds to many promoters and enhancers to regulate transcription. For instance, transcription of *TP53* is dependent on ARID1A [324]. Note that mutations in *ARID1A* and mutations in the *ARID2*, encoding the component of the PBAF complex, occur recurrently as somatic alterations in MM (see Section 3). Waller et al., identified another candidate gene, *USP45,* as associated with a familial risk of MM [319]. This gene is particularly interesting since it encodes ubiquitin hydrolase that controls the activity of ERCC1, a subunit of the ERCC1–ERCC4 endonuclease (the ERCC1–XPF complex), a core component of nucleotide excision repair that participates in CSR [325,326].

Another gene linked to MM development, *DIS3*, is also one of the top somatically mutated genes in MM (see Section 3) [28,53,82,86,327,328,329]. The presumptive loss-of-function mutations in *DIS3* have been reported in about 2.6% of familiar cases with MGUS and MM [330]. *DIS3* encodes for the catalytic 3′-5′ exonuclease subunit of the exosome complex, which is implicated in processing RNA transcripts and regulating mutagenesis and recombination at the *IGH* locus [331,332]. It is tempting to speculate that *DIS3* mutations might be implicated in aberrant CSR, leading to primary genomic rearrangements at the *IGH* locus (see Section 5.2 for more discussion).

WES analysis of the family with increased prevalence of MGUS and MM allowed identifying a missense mutation in the *EP300,* encoding transcriptional coactivator p300, as a likely causative variant [333]. Recurrent somatic mutations in *EP300* in MM were also described (see Section 3). In addition to that, rare germline variants associated with MM familiar cases were reported for *CDKN2A* [334,335]. *CDKN2A* encodes for several transcripts: p16-INK4A and p14ARF encoding for proteins acting as tumor suppressors for different cancers. The p16-INK4a belongs to the INK4-family of specific inhibitors of CDK4/6 kinases, controls G1-to-S progression, and is an essential component of cellular senescence program [336,337]. Another transcript, p14ARF, is a result of alternative splicing, and the synthesized protein plays a role in *TP53* activation by binding to MDM2 [338,339]. Methylation of the regions corresponding to p14ARF and p16-INK4A could represent early events in the pathogenesis and development of plasma cell disorders and were reported in MM [340,341]. Expression of p16-INK4A is often absent in MM [342].

It should be noted that *LSD1*, *ARID1A, USP45, DIS3*, and *EP300,* in addition to the mechanisms discussed above, can be implicated in the telomere maintenance pathway, and thus might influence the predisposition to MM through telomere structure and length regulation (see discussion in Section 6).

More than 100 different SNPs were also reported in GWAS studies to associate with the risk of MM development [343,344,345,346,347,348,349,350,351] or MGUS [352,353]. Genes and pathways affected by these SNPs are proposed to be susceptibility candidates based on linkage disequilibrium distance and/or altered gene expression. In many cases, they affect non-coding regions or lie thousands of base pairs away from the candidate gene. In total, genes affecting several biological pathways have been tightened to MM development risk, including histone modification and chromatin remodeling, transcription, and co-transcriptional RNA maturation, *IRF4-MYC* regulatory network, B-cell differentiation, genome stability, and telomere maintenance (see Table S3). A recent approach taking advantage of a transcriptome-wide association study allowed for the expansion of GWAS analysis and identified new MM risk genes, including DNA/RNA-editing cytosine deaminases *APOBEC3C*, *APOBEC3D*, *APOBEC3F*, *APOBEC3G,* and *APOBEC3H* at 22q13.1, responsible for immunity, and *RNF40* at 16p11.2, involved in DSB-repair [354].

It should be noted that some SNPs identified in GWAS studies were linked to chromatin remodeling and epigenetic regulation that, in connection with the risk variants observed for family cases, highlights the importance of chromatin organization and regulation for MM development. One example is SNPs at 7q36.1, presumably affecting the expression of *SMARCD3*, encoding a component of the BAF complex [344,346,347,355]. Several other nucleotide variants affect components of the PRC1 and PRC2, the two major Polycomb group (PcG) repressive complexes controlling cell-specific transcriptional programs via histone modifications [356,357]. Thus, SNPs at 22q13.1 localize to the *CBX7* locus encoding a component of PRC1, the complex that facilitates H2AK119 ubiquitination and plays a role in lymphogenesis and hematopoietic malignancies [345,358,359,360]. H2AK119 ubiquitination at sites of DNA breaks has been linked to DSB repair [357,361]. Overexpression of *BMI1*, encoding for another subunit of the PRC1 complex that stimulates the ubiquitinase activity of PRC1 toward H2AK119 and promotes DSB repair, has been observed in myeloid malignancies [361,362]. BMI1 is essential for MM cell growth, and its depletion sensitizes MM cells to bortezomib [363,364]. It worth noting that another SNP, rs34229995, associated with MM localizes to the regulatory region of *JARID2* encoding the subunit of the PRC2 complex [344,365]. JARID2 binds to H2AK119Ub and facilitates trimethylation of H3K27 by the PRC2, thus promoting transcriptional repression [357]. Curiously, PcG complexes are known negative regulators of *CDKN2A* expression, the gene that has been linked to familiar MM cases [366,367]. The involvement of *CDKN2A* in MM development is additionally confirmed by the association of rs2811710 located in the regulatory region of the *CDKN2A* with MM risk [344]. Several SNPs at 7p15.3 are linked to the increased expression of *CDCA7L* [352,368,369,370]. *CDCA7L* affects the genome-wide methylation level, and it is a target gene of MYC; it is also involved in *CCND1* upregulation in glioma [371,372].

Another group of SNPs associated with MM development is linked with transcription regulation and RNA processing. Thus, rs4325816 is located in the intron of the *SP3* gene encoding transcription regulator, which is among the bortezomib targets [373]. SNPs located at 19p13.11 are located near the *KLF2* gene, which is listed among hotspots for structural variations in MM [83]. KLF2 is a transcription factor that plays a role in maintaining B-cell quiescence and regulating IRF4, BLIMP1, and AID levels; its expression is induced after pre-BCR signaling and is maintained until B-cell activation [374]. Bortezomib upregulates *KLF2* [375]. SNPs at 5q15 are linked with *ELL2* encoding for the elongation factor of RNA polymerase II, which regulates immunoglobulin mRNA processing in plasma cells [376,377,378]. ELL2 travels with the RNA polymerase II across the *IGH* μ- and γ-gene segments and mediates the association of polyadenylation-cleavage factor CstF-64 with the RNA polymerase II and switching to a secretory-specific poly(A) site [378].

Regulation of DNA repair and B-cell-specific genome rearrangements, such as CSR and V(D)J recombination, seem to associate with MM development. Polymorphisms in the *AICDA* gene were linked with better outcomes in MM patients [379]. Decreased MM risk has also been reported for SNPs located near *LIG4*, encoding the DNA ligase IV essential for V(D)J recombination [379,380]. On the other hand, SNPs located in the regulatory regions of *XRCC5* encoding Ku80 and *XRCC4* encoding a factor associating with DNA ligase IV conferred susceptibility to MM [381]. A missense mutation in the *RFWD3* encoding a RING-type E3 ligase that belongs to a FA group and is essential for HR repair, replication fork restart, and translesion DNA synthesis predisposes to MM [344,382,383,384,385,386]. Several risk SNPs associate with *TNFRSF13B* and *TOM1,* the genes implicated in immunoglobulin class-switching regulation [343,344,368,377,387,388]. Autophagy and intracellular trafficking play an important role in plasma cell differentiation and immunoglobulin production; consistently, the defects in autophagy have been linked to MM development [216,344,389]. It should be noted that autophagy-deficient Atg5−/− plasma cells have higher expression of *BLIMP1* and *IGH* genes [389,390]. Several SNPs affect genes with the proposed centrosomal role, including the *ULK4* and *CEP120,* and might potentially impact chromosome stability [348,368,391,392,393]. In addition to the discussed loci, multiple SNPs affecting genes associated with the telomere maintenance pathway have been linked to MM predisposition risk (see Section 6 for discussion).

Altogether, the set of risk mutations and SNPs associated with MM predisposition highlight the role of chromatin structure and epigenetic regulation, DNA repair, transcription, and RNA processing, as well as B-cell-specific processes necessary for antibody synthesis and maturation as significant factors influencing disease development. It is plausible that B-cell-specific *IGH* locus reorganization might predispose to certain types of chromosomal rearrangements and aberrations that initiate MM development. It should be noted that V(D)J and CSR are mutagenic processes per se and might initiate a cascade of genomic alterations, ultimately leading to carcinogenesis (see Section 5.2 for more discussion).

## 3. Mutational Landscape of MM and Its Precursors

Complexity and heterogeneity are hallmarks of the MM cells’ genomic landscape. The distribution of mutation loads is highly variable in both MM and other hematological tumors. However, MM (as well as other B-cell neoplasms) tend to possess more point mutations than other hematological malignancies, though less than cancers associated with higher mutagenic exposures, for example, melanoma, which has roughly 10 times more mutations compared to MM [394]. The first NGS studies of MM focused on detecting mutational profiles. The scope of the following studies was expanded to identify specific genetic alterations for different stages of the disease, find driver mutations, deduce MM-specific mutational signatures, and locate pathways involved in the progression from early stages toward MM (Table S1). The tumor-normal pair approach compares the genomes of tumor and germline variants and allows for finding mutations present in MM only. 

A total of about 60 significantly mutated genes (i.e., the genes that are mutated more often than expected by chance, as determined by a specific algorithm) have been identified in MM cells [28,61,81,82,86,328,395,396]. Most mutations affect the following pathways: signaling RAS/MAPK, NF-kB, MYC, DNA repair, RNA interaction, chromatin regulation and epigenetic mechanisms, transcription, cell cycle regulation, plasma cell differentiation, cellular adhesion, and motility. Genes recurrently mutated in MM and the corresponding pathways implicated in MM development are summarized in Table S2.

Mutations affecting the RAS/MAPK pathway prevail in newly diagnosed patients and were found in 19–50% of patients [28,81,82,86,328,397,398]. Mostly, *KRAS*, *NRAS*, and, to a lesser degree, *BRAF* mutate in different clones. *KRAS* and *NRAS* mutations are nearly always mutually exclusive but can coincide with *BRAF* mutations [28,328]. These events can be both clonal and subclonal, meaning they can arise early and late in clone evolution, correspondingly [28,328]. Mutations in other genes, such as *NF1, PTPN11,* and *RASA2*, leading to activation of the RAS/MAPK pathway, were also reported [86,328,398] (Table S2). The NF-κB pathway participates in apoptosis, differentiation and proliferation of cells, immunity, inflammation, and lymphopoiesis, is affected in 40% of MM cell lines and 12–20% of MM patients, and is represented by mutations mostly in *TRAF2, TRAF3, CYLD, NFKB2*, *NFKBIA,* and *LTB* genes [28,82,86,328,399,400]. Despite their high prevalence in MM and apparent association with tumor progression (i.e., driver mutations), mutations in RAS/MAPK and NF-kB pathways in many cases showed no prognostic impact on survival [82].

Mutations in some of the DNA repair pathway genes are also frequently found in MM patients. In most cases, they affect genes encoding factors facilitating DNA damage sensing: the p53 tumor suppressor and the ATM/ATR kinases facilitating p53 phosphorylation [82,86,328,401]. Frequency of detected *TP53* changes correlated with the disease stage: On average, 5–8% of MM patients carry *TP53* point mutations at the diagnosis, but up to 25% at the advanced stages of the disease [402]. *HUWE1* encoding E3 ubiquitin ligase involved in DNA damage response and DNA repair is mutated in a significant cohort of MM patients, contributing to tumor growth and MM treatment [86,403]. Mutation in other DNA repair pathways, e.g., HR, FA/BRCA, NER, BER, and MMR, occur in MM patients more rarely, with a frequency of about 1% or less [82,86,404,405,406]. As was discussed in Section 1.2, mutations in some of these genes or deregulation of their expression may associate with resistance to chemotherapeutic drugs, e.g., melphalan [160,404].

Mechanisms of RNA processing have a significant impact on MM development. The *DIS3* gene is found recurrently mutated in MM patients with a frequency of about 8–11% at the time of diagnosis [28,53,81,82,86,327,328,329]. *DIS3* mutations are also associated with inherited MM risk (see Section 2). Somatic *DIS3* mutations in MM are mostly clonal; however, their subclonal accumulation has also been noted and has been associated with shorter overall survival [28,327,328]. Another significantly mutated gene in MM is *FAM46C,* which is affected in 5–13% of cases and is deleted in 20% of patients [29,81,82,86,407,408]. *FAM46C* encodes for a non-canonical, poly(A)-polymerase controlling mRNA stability and facilitating B-lineage-specific onco-suppressor functions [409,410]. *FAM46C* is upregulated during plasma cell development and contributes to immunoglobulin heavy and light chains’ mRNA poly(A) length, stability, and immunoglobulin production [409,411,412]. Mutations in *FAM46C* are specific to MM and are infrequent in other cancers. They are associated with worse overall survival in MM patients and are predominantly found in hyperdiploid tumors [29,328,407]. A minor proportion of MM cases harbor mutations in the other genes encoding for RNA interacting proteins [29,398,406] (Table S2). Mutations in the *RPS3A* are common for hematological malignancies [413]. In addition, mutations in the genes encoding for ribosomal proteins RPL5 and RPL10 have been noted and *RPL5* is frequently deleted in MM [414].

Mutations affecting chromatin regulation and epigenetic mechanisms are common for MM. One interesting group is represented by genes encoding linker histones. Missense mutations clustered in the regions corresponding to the highly conserved globular domain of the linker histones encoded by genes *HIST1H1B, HIST1H1C, HIST1H1D, HIST1H1E*, *HIST1H2BK*, and *HIST1H4H* were found in MM cells [61,82,86,398,415]. Linker histones facilitate nucleosome–nucleosome contacts and contribute to the formation of nucleosome clutches representing higher-order chromatin interactions. They also play a role in establishing Topologically Associated Domains (TADs) and modulation of chromatin topology within the nucleus [416]. Affecting linker histones’ function can have an impact on chromatin structure, genome stability, and gene expression, which ultimately may influence the rate of genetic and epigenetic changes in MM.

Another group is represented by genes encoding histone modifiers. Genes of histone lysine methyltransferases and demethylases frequently alter in MM, and their mutations may account for epigenetic deregulation associated with disease progression and resistance to therapy [417]. Most frequently, mutations are observed in the genes of the *MLL* family, *MLL (KMT2A), MLL2 (KMT2B), MLL3 (KMT2C), MLL4 (KMT2D),* and *MLL5 (KMT2E),* involved in methylation of the H3K4 residue, a mark of transcription activation [81,86,415]. Other mutated methyltransferase genes include, but are not limited to, *EHMT2, SETDB1,* and *SETD2,* which contribute to methylation of H3K9, H3K27, H3K36, and *MMSET*, which contribute to methylation of H3K27, H3K36, and H4K20 [29,86,415]. *MMSET* is a master regulator of many pro-oncogenic pathways, and its upregulation or gain-of-function mutations have important prognostic significance (see Section 1.2) [418,419]. Mutations affecting demethylases are most frequently found in *UTX* (*KDM6A*), *KDM3B,* and *KDM5C,* which encode enzymes removing methyl residues from methylated H3K27, H3K9, and H3K4, correspondingly. These methylated sites are considered marks of silent or inactive chromatin [29,86,328,329,415,420]. Moreover, MM genomes harbor mutations in the genes *EP300* and *CREBBP(CBP)* [86,415]. *EP300* and *CREBBP* are closely related paralogs encoding major lysine acetyltransferases in metazoans, functioning as transcriptional coactivators and tumor suppressors [421]. The p300 contributes to the acetylation of the H3K27 and H3K18 histone residues, the modifications that associate with active promoters and enhancers. In addition to that, p300 acetylates many non-histone targets. Mutations in *EP300* are associated with many different cancers and are often found in patients with myelodysplastic syndromes [421,422]. Chemicals that bind to bromodomains of p300 and CBP abrogate the viability of MM cell lines by interfering with the *IRF4* transcriptional program [423]. Mutations in *NCOR1*, encoding a co-repressor recruiting and activating HDAC complexes, were also found [53,86].

Chromatin remodelers participate in the regulation of chromatin organization essential for DNA replication, DNA repair, transcription, and other processes and play an important role in genome stability. It is not surprising that MM genomes also possess mutations in the genes encoding chromatin remodelers and their interacting partners, including *CHD2, CHD4, ARID1A*, *ARID2, ATRX,* and *ZFHX4* [9,82,86,415]. Noteworthy, ARID2 is a neosubstrate of CRL4^CRBN^ induced by IMiDs, and it is effectively targeted for degradation by pomalidomide [424]. Mutations in the *ARID1A* also associate with the risk of MM development (see Section 2), highlighting the role of chromatin regulation in shaping the MM genome.

A special niche is taken by genes encoding DNA modifiers. *IDH1, IDH2*, *DNMT3A* encoding DNA methyltransferases and *TET2* (Ten-Eleven Translocation 2) encoding for methylcytosine dioxygenase are among frequently mutated genes in MM [60,86,415]. The DNA modification process seems to play an essential role in transitioning from MGUS to MM. A global transition to CpG islands’ hypomethylation in genic regions has been noted after a shift from a non-malignant state to MM. The event affected the expression of many genes and was linked to the downregulation of the *DNMT3A* [425,426,427]. The hypermethylated regions in MM cells mainly concentrate in B-cell-specific regulatory regions (see Section 5) [428]. A correlation between different structural rearrangements in MM and methylation patterns has also been described [425,429]. Interestingly, a global level of a 5-hydroxymethylcytosine mark is also decreased in MM compared to normal plasma cells [430]. TET2, which facilitates oxidation of methylcytosine in the DNA, thus contributing to the production of 5-hydroxymethylcytosine, is frequently deregulated in myeloid malignancies, and *TET2* inactivation has been proposed to play an initiating role in age-related hematological cancers [422,431,432,433]. Importantly, TET enzymes bolster the expression of *AICDA,* thus augmenting CSR [434]. Both *DNMT3A* and *TET2* mutations are enriched during the relapse stage of MM, which correlates with chemo-resistance [415].

Among genes encoding transcription factors, frequent mutations in MM have been observed in *IRF4, BLIMP1(PRDM1)*, and *XBP1* [28,61,81,82,86,328,398]. As discussed above, BLIMP1, XBP1, and IRF4 are transcription factors expressed in antibody-secreting plasma cells and are important for plasma cell commitment (see Figure 3). Mutations in the cis-regulatory elements of other important players of B-cell development, e.g., *PAX5, BCL6,* and *PAXIP1* (PTIP)*,* were also described in MM and may affect their expression [60,398]. Other transcription factor genes whose mutation are found in MM are *EGR1, MAX, ZNF208, MAF, MAFB, IKZF1,* and *IKZF3* [28,29,61,81,82,86,328,398]. *EGR1* mutations are associated with hyperdiploidy and often occur in WRCY DNA sequence motifs, implicating SHM and AID as causative factors [28,82,435]. Mutations in *MAX*, *IRF4*, and *EGR1* may affect the expression level of the *MYC* oncogene. Despite these facts, *IRF4* and *EGR1* mutations positively impact the survival of MM patients [82].

Several cell cycle pathway genes mutate recurrently in MM. The most essential are *CCND1, CCND3, RB1, CDKN2C*, and *CDKN1B* [28,60,61,82,86,161,395]. Mutations in *CCND1* are an early event in the development of MM and associate with t(11;14) translocation (see Section 5.1). *CDKNK2C* and *RB1* are potent cell-cycle regulators, and their inactivation correlates with a high proliferation index.

Dynamic changes and mutational processes involved in MM pathogenesis are revealed by analysis of genomes at different stages of the disease, starting at diagnosis, progression, and relapses or post-treatment time points [29,61,63]. The genetic determinants of the MM evolution from MGUS and SMM stages (Figure 1) are much less understood. Several WES and WGS studies characterized the spectrum of mutations and structural rearrangements of MGUS and SMM to find the genetic patterns of progression from premalignant stage to MM [20,48,51,62,436,437,438,439]. The genomic dataset of both MGUS and SMM contains fewer mutations than MM, but mutations of *KRAS, NRAS, DIS3, HIST1H1E, EGR1*, *LTB,* and *CCND1* occur both in MGUS and MM [20,51,52,62]. The mutational landscape of SMM is similar to newly diagnosed MM, although it varies in groups with different progression risks to MM [48,62]. Mutations of *NRAS, KRAS, BRAF, DIS3, FAM46C, TRAF3, TP53, ATM, LTB, EGR1, RB1, MAX, CDKN2A,* and other genes were found in SMM patients [47,48,51,56]. Importantly, no mutations in the DNA repair genes such as *TP53, ATM,* and *ATR* were identified in stable MGUS patients, and biallelic inactivation of *TP53*, *RB1*, *DIS3*, *MAX,* and *CDKN2A* were rare events for low-risk SMM, suggesting that these abnormalities are associated with tumor progression [48,51,52,62].

Several studies have revealed genomic changes in response to drug therapy [25,53,54,60,440,441,442,443,444]. As was discussed in Section 1.2, the CRBN protein is the main target of IMiDs, such as thalidomide, pomalidomide, and lenalidomide, used in MM therapy. Mutations in the *CRBN* and in the genes acting in this pathway, such as *IKZF1, CUL4A*, and *IRF4*, were more frequently found in patients refractory to IMiDs and associated with worse outcomes [329,441,443]. A shorter duration of therapy with IMiDs was proposed to result in fewer mutations in cereblon pathway genes [209,443]. PIs are widely used in many MM treatment regimens, but almost all patients develop resistance to these drugs over time. It has been shown that treatment with PIs may cause accumulation of mutations in the genes encoding components of proteasome such as *PSMB5*, *PSMB8, PSMD1*, and *PSMG2* [442]. Recent studies suggest that MM at relapse has a more complex genetic landscape compared to primary tumors and highlights the biological role of *TP53* inactivation and certain structural genome rearrangements (e.g., 1q gain, del(17p), and *MYC* translocations, see Section 5.1) rather than mutations in genes associated with resistance to PIs or IMiDs in acquired resistance to therapy [25,54,60]. Notably, the response to IMiDs could also be influenced by the naturally occurring polymorphism in the *CRBN* gene region and by the epigenetic modifications of the *CRBN* regulatory elements [445,446].

## 4. Mutational Signatures in MM Genomes

**Figure 4:**
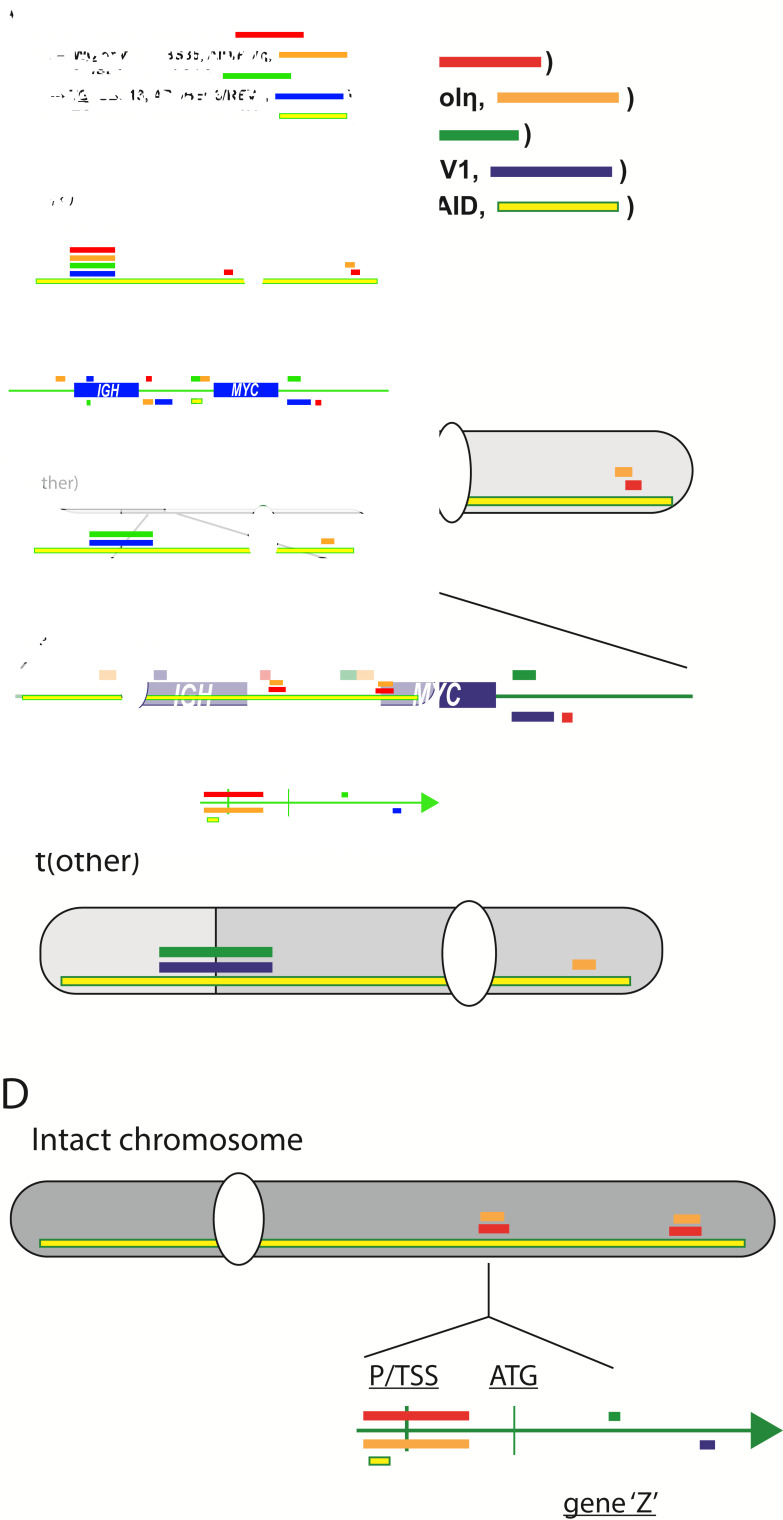
Localization of regions with specific mutational signatures in the multiple myeloma genomes. Mutational signatures identified in MM genomes are shown on panel (**A)**. Panels (**B**–**D**) schematically show locations of these signatures on the chromosomes. There is substantial variability in the types of translocations and the strength of each individual mutational signature in the individual patient genomes and within tumor subclones. Thus, the scheme represents “averaged” features of the mutational signatures. Deamination in CpG motifs (yellow boxes with green border) is scattered randomly in the genomes. Translocations involving the *IGH* locus can possess all four deaminase-related mutational signatures found in myeloma genomes. An example of translocation involving the *IGH* and *MYC* loci is shown (**B**). Both AID- and APOBEC3-related mutations can be found around the breakpoint. As with other translocations (panel **C**), APOBEC3-related mutations (green and blue) are happening due to the deamination of ssDNA resulting from the resection of double-stranded breaks. The amount of resection, the lifetime of resected ends, and availability of APOBEC3 proteins in the nucleus at the time of the translocation event should affect the length and density of the hypermutated regions. Clusters can span over Mb distances around the breakpoint, with most dense regions having inter-mutational distance less than 1 Kb. AID-related mutations (red and orange) are found not only in the *IGH* locus but also scattered throughout the genome, often found in the 5’-non-coding regions and generally in the beginnings of the genes (**B**–**D**). This is a result of the off-target effects of SHM. These clusters are generally smaller than APOBEC3-related clusters. Genes may possess one of the AID-related signatures or both. Gene bodies are more likely to possess APOBEC3-related mutations (**D**). This effect occurs because, even though deamination of 5’-regions of the genes is a natural feature of all APOBEC proteins, there are no known trans-acting factors capable of recruiting APOBEC3 enzymes to the beginning of the genes or switch regions of the *IGH* locus, as happens in cases of AID in SHM and CSR. This scattered signature of APOBEC3 in the genome is different from classical *kataegis* in that there are no significant and dense mutational clusters and is more reminiscent of “omikli”, which has been recently described in other cancers [455].

Tumor cells accumulate multiple mutations during cancer initiation and progression. Types of mutations, DNA sequence context where they happen, and the distribution of mutations in the genome help deduce what molecular processes operated during tumor evolution [447,448]. It is clear that the mutational landscape of a tumor is shaped by the concerted action of many factors, including therapeutic drugs: There are signatures associated with age, smoking, UV light, various DNA repair defects, APOBEC deaminases, chemotherapy, and others [448]. At least five signatures are seen in MM (Figure 4). High-dose chemotherapy contributes to the mutational landscape of MM, increasing the mutational burden and producing new mutational signatures [54,59,63,145,146]. Thus, melphalan signature has been found in the transcribed strand implementing transcription-coupled NER on damaged DNA [63,146]. Three mutational signatures common in cancer genomes were attributed to the action of the AID/APOBEC family of cytosine deaminases [449]. AID/APOBEC is a superfamily of enzymes that convert cytosine to uracil and consists of two subfamilies, AID and APOBEC3 [450]. AID (activation-induced cytosine deaminase) edits DNA in immunoglobulin genes in the process of SHM and CSR in the maturating B-cells. Proteins of the APOBEC3 subfamily mediate the restriction of viruses by deaminating viral cDNA. AID/APOBEC proteins are active on ssDNA and can act processively, thus generating clustered mutations [451]. Individual APOBEC protein family members possess intrinsic deamination sequence specificity, thus allowing for attributing the mutations found in cancers to the action of specific APOBEC based on the sequence context of the changes. It has been proposed that AID/APOBEC processivity and availability of long ssDNA regions in tumor evolution give rise to a phenomenon called *kataegis*–localized hypermutated regions [452,453,454].

Signatures of AID/APOBEC deaminases-related mutagenesis are found in many cancers, including MM [63,79,82,394,395,435,447,448,456,457,458]. The first paper on the classification of mutational signatures in cancer [394] reported two signatures in multiple myeloma: signature 2, which is attributed to the activity of APOBEC3 proteins, and signature 5, which has an obscure mechanism, possibly related to smoking. Further in this review, mutation signatures are referred to as SBS (for Single Base Substitutions), according to the newest classification [448] (see https://cancer.sanger.ac.uk/signatures/sbs/, accessed on 20 October 2021). One of the early papers also reported detecting clustered mutations, *kataegis*, in the APOBEC3-specific sequence context in MM [459]. AID preferentially deaminates cytosines in WRCY sequence context (mutated base underlined), where W = A/T, R = A/G, and Y = C/T. The resulting uracil in DNA (WRUY) can be replicated through emergence of C->T transitions and WRTY (AID SHM signature, SBS84) [448] or by inducing a series of DNA repair events involving error-prone DNA polymerases η and θ, leading to A->C/G substitutions in WA motifs (SBS85, Figure 4) [435,460,461,462]. In the hematological malignancies’ literature, SBS85 is often referred to as a non-canonical AID signature [63,398].

Proteins of the APOBEC3 subfamily have a strong preference for TC sequences, thus causing TC->TT and TC->TG mutations (the latter likely due to the activity of REV1 translesion DNA polymerase) [463], giving rise to signatures SBS2 and SBS13 (Figure 4). These signatures are often referred to as “APOBEC” signatures, but they are different from the above AID signature, a member of AID/APOBEC family enzyme (but different subfamilies). With the use of a slightly longer sequence context, it is possible to separate SBS2 and SBS13 signatures into APOBEC3A- and APOBEC3B-specific signatures [448,464,465].

One of the first reports on whole exome/genome sequencing (WES and WGS) of MM revealed a higher mutation frequency in noncoding and intronic regions, as compared to protein-coding regions, which was at least partially explained by the action of transcription-coupled repair [81]. In addition, 5’-UTRs and the beginning of the coding sequences of genes known as SHM targets (immunoglobulin genes) and off-targets (genes that do not normally undergo SHM, but tend to be mutated in cancers due to abnormal AID recruitment, for example, *BCL6* and *MYC*) were also enriched with mutations [81,466]. Mutations at the beginning of the genes (encompassing both promoter regions and beginning of CDSs) were found in the typical AID consensus motif WRCY, implicating that AID was a mutator in studied MM cases [28].

The following WES and WGS studies of MM elaborated on the original findings. Using WES, signatures attributed to three mutational processes have been detected in MM: (1) APOBEC3-specific signature, sometimes with mutation clustering, *kataegis*; (2) SHM-like mutations (in C-T pairs and A-T pairs), often clustered; and (3) C->T mutations in CpG motifs, age-associated signature found in many cancers that are caused by deamination of methylated cytosine, which is generally considered to be spontaneous, but can also be AID-mediated [29,456,467]. Clusters of mutations were often found close to translocation breakpoints, with AID-type mutations generally found in translocations involving the *IGH* locus, the natural target of SHM, while other translocations sometimes possessed APOBEC3-like mutations [456]. More recent reports involving WGS allowed for the characterization of MM genomes with improved resolution. Signatures of APOBEC3′s, AID, and SHM-Polη (“non-canonical AID”, SBS85) have been found, with the *IGH* locus possessing *kataegistic* regions containing all three mutational signatures [47]. Similar results were obtained in a study combining WES and WGS, and more AID-type mutations have been found in the driver genes, indicative of the undergoing selection [395]. The combined results indicate that SHM-like, AID-initiated mutational events usually happen in the early stages of myeloma evolution when B-cells undergo antigen-dependent maturation in GC [63,468,469]. Later in myeloma evolution, APOBEC3-induced mutagenesis predominates. Interestingly, myelomas with specific translocations (t(14;16)/*MAF*, t(14;20)/*MAFB,* and t(8;14)/*MAFA* the “maf” translocation group, see Section 5.1) possessed an overall higher number of mutations and a larger proportional contribution of APOBEC3 signature compared to tumors with other translocations [63,395,398,456]. It turns out that the overproduction of transcription factors MAF, MAFB, and MAFA as a result of translocations in these tumors leads to the increased expression of APOBEC3s, which in turn causes increased APOBEC3-dependent mutagenesis [456]. Moreover, by separating APOBEC3A and APOBEC3B mutational signatures [464], Rustad et al. [63] concluded that APOBEC3A, but not APOBEC3B, is the main source of mutations in these highly mutated “maf” translocation type myelomas. Altered relative proportions of these mutational signatures in tumors with higher APOBEC3A-to-APOBEC3B ratio reinforced these findings [62]. Overall, the increased APOBEC3 activity is more frequent in the progressive disease. However, stable MM precursor clones may possess “maf” translocations and have an increased APOBEC3A:3B ratio [62,470]. Clearly, more research is needed to decipher the relation between “maf” translocations, APOBEC3A, APOBEC3B, and MM development. Further studies using more sophisticated algorithms of separating highly similar mutational signatures [465] might further clarify these complex processes.

Taken together, MM genomes’ sequencing data combined with analysis of specific mutational signatures favor a model where AID-dependent SHM machinery participates early in MM evolution, inducing mutations in driver genes and leading to a bias towards noncoding regions and the beginnings of the genes. Later, APOBEC3-dependent mutagenesis is continuing to shape tumor genomes, with the age-related CpG deamination process stably contributing during the whole process of tumorigenesis [63]. Finally, APOBEC-induced mutagenesis is associated with MM progression from its precursors [48,51,62]. It has been proposed that testing for APOBEC activity at the time of diagnosis might suggest better, more personalized treatment options [438]. Specifically, since MM patients with high APOBEC activity have a poor prognosis, it was suggested they might benefit from more aggressive treatments. It is worth noting that approaches are being developed to target APOBEC enzymes [471].

## 5. Structural Variations in the MM Genomes

### 5.1. Driving Genomic Rearrangements in MM

MM is characterized by karyotypic changes in the malignant plasma cells. Fluorescent in situ hybridization along with classic cytogenetic analysis has been used for years to analyze the karyotype in MM [472,473,474,475]. Recent advances in NGS sequencing of tumor genomes allowed more precise and accurate characterization of the genomic landscape in MM, determination of complex rearrangements, and tracking the chronology of events [79]. Various types of karyotype instability observed in MM include chromosomal gains or losses, translocations, and complex genomic rearrangements [78,476]. Karyotype instability is currently viewed as an early event in the development of MM, driving the development of the disease, and represents an important prognostic factor. Two major classes of primary karyotypic changes in MM are hyperdiploidy and *IGH*-translocations.

Trisomies of several odd chromosomes, most often chromosomes 3, 5, 7, 9, 11, 15, 19, and 21, are found in tumor cells in approximately 60% of patients with MM, and this is referred to as a hyperdiploid state [61,477,478]. The type and number of trisomies have prognostic significance [479]. Recent data suggest that the acquisition of the hyperdiploid state is a stepwise process, which begins at the early stages of the disease development [61,79,478]. Plasma cells in most patients with MGUS carry numerical abnormalities of at least one of chromosomes (most often 6, 9, 13, 15, 17, 19), and up to ~50% are classified as hyperdiploid [57,478,480,481]. More chromosomes are gained as the disease progresses, and some patients acquire whole-genome duplications at the extreme end of the disease evolution [61,478]. The most common chromosomal translocations in MM involve chromosome 14 and specifically occur at the *IGH* locus at 14q32.33. This locus encodes for heavy immunoglobulin chains and undergoes multiple changes during B cell differentiation, apparently providing an important source of genomic instability in MM. The chromosomal aberrations at the 14q23.33 are regarded as early stages of MM development as they are found in nearly 50% of patients with MGUS and SMM [57,482]. Five major types of chromosomal translocations involving *IGH* in MM are t(4;14), t(6;14), t(11;14), t(14;16), and t(14;20) [483,484]. In all these cases, a specific oncogene on the partner chromosome is placed under the *IGH* enhancer control, resulting in an upregulation of its expression. Translocations t(11;14) and t(4;14) are the most frequent and usually are found in 15–20% of patients with MM each, while t(14;16), t(6;14), and t(14;20) are less common and each occur with a prevalence of 5% or less.

Translocation t(4;14) involving the 4p16 region is associated with adverse prognosis and results in upregulation of *FGFR3* and *MMSET* genes in most carriers [78,483,485,486]. This translocation is hard to identify by G-banding because the breakpoints on chromosome 4 occur in the telomeric region close to the *FGFR3* that becomes associated with the 3′ enhancer(s) on derivative chromosome 14 [484]. On chromosome 14, breakpoints usually map to the switch regions that play an important role in immunoglobulin isotype switching (see Section 5.2), indicating that the mechanism of this translocation is connected to CSR [484]. *FGFR3* overexpression results in the activation of the transcription factor STAT3 and consequent upregulation of its target *BCLXL* gene, which is functioning in suppressing apoptosis and promoting MM development [487]. Besides *BCLXL*, STAT3 regulates the expression of other anti-apoptotic genes, *MCL1* and *BCL2,* key oncogenes including *MYC* and *CCND1*, and telomerase reverse transcriptase *TERT* [488,489]. Interestingly, although mutations in *STAT3* have not been reported in MM, STAT3 has been found to be constitutively activated in plasma cells isolated from MM patients as well as in MM cell lines [490,491,492]. In addition to that, IL6-STAT3 signaling is a key regulator of the B-cell differentiation program, and STAT3 is specifically required for the transition to antibody-secreting program [214,493,494]. *FGRF3* is also frequently found mutated in primary MM tumors and MM cell lines carrying t(4;14), which also may lead to STAT3 and MAPK activation [495]

Upregulation of *MMSET* leads to an increase of active chromatin mark H3K36me2, decrease in the repressive mark H3K27me3, and global increase in chromatin accessibility [418,419,496]. MMSET mediates p53 degradation and it is involved in the regulation of expression of DNA repair genes; it enhances DNA damage repair, cell proliferation, and resistance to melphalan [497,498]. MMSET regulates DNA damage-induced histone H4K20 methylation, 53BP1 foci formation, and DSB repair [499]. It also interacts with PCNA, and it is required for replication licensing and normal S-phase progression [500]. MMSET promotes NHEJ at unprotected telomeres and, therefore, might contribute to the generation of complex genomic rearrangements [501]. MMSET also contributes to AID-mediated DSBs at switch regions at the *IGH* locus [502].

Translocation t(11;14) attaches the cyclin D1 gene *CCND1* to the *IGH* enhancer. On 11q13, the breakpoints are dispersed over 330-kb intervals centromeric to the *CCND1* gene [484].

Translocation t(6;14) involving 6p21 juxtaposes cyclin D3 *CCND3* gene to the *IGH* enhancer. The breakpoints are within 150-kb intervals centromeric to the *CCND3* [484].

Several primary IGH translocations form the so-called “maf” group because they result in upregulation of the MAF transcription factors. Translocation t(14;16) juxtaposes the *IGH* locus to the 16q23 with breakpoints located in the last exon of the tumor suppressor *WWOX* and affects the neighboring oncogene *MAF* expression. Importantly, *WWOX* is located in the same band 16q23.2 with a highly unstable region, a common fragile site (CFS) FRA16D [503]. FRA16D CSF is known by association with genomic instability in different types of cancer, and *WWOX* downregulation is thought to play a role in tumor development [503,504]. A translocation involving 20q12, t(14;20), results in the upregulation of the *MAFB* gene, a paralog of *MAF* [505]. The third translocation belonging to the “maf’ group, t(8;14), juxtaposes the *IGH* locus to another paralog of *MAF*, the *MAFA* gene, at 8q24 and it is rarely found in newly diagnosed MM patients [506]. These translocations are characterized by high APOBEC activity since MAF factors regulate APOBEC expression [63,456] (see Section 4). A few other translocations involving the *IGH* locus in MM involve 1q21, 6p25, 20q11, 21q22, and 22q12 and are infrequent in primary patient samples; they are mostly found in cell cultures [484,506,507].

Significant association of translocations with increased mutagenesis in certain genes has been observed. For instance, t(11;14) is associated with mutations in *CCND1* and *IRF4,* t(4;14) group is associated with mutations in *FGFR3*, *PRKD2*, and *DIS3*, t(14;16) coincides with mutations in *MAF*, *ATM*, *BRAF*, *TRAF2*, *EP300,* and *DIS3*, and t(14;20) accumulates mutations in *MAFB* [29,61,81,82,86,395,456]. The genes juxtaposed to the *IGH* regulatory regions because of rearrangement may harbor cluster mutations as a result of SHM driven by AID or other factors spreading to new genomic sites. It is also possible that new genomic locations and increased expression of the affected genes may contribute to the mutation rate. Note that some of the listed genes are not affected directly by the translocations and other mechanisms can account for accumulation of mutations in these regions. Interestingly, the role of *IGH* enhancers in plasma cell development and oncogenesis has led to an idea about the possibility of regulating their activity through small molecules [508]. One of the selected molecules in this research inhibited the expression of the translocation-induced oncogenes and showed efficacy in trials with MM cell lines.

Other events that can be tracked as early as the MGUS state include a gain of 1q, 13q deletion, and, in rare cases, 17p deletion [20,57,61,478,509]. Some karyotypic abnormalities detected in MM, hyperdiploidy, or a few types of *IGH* translocations have a neutral or neutral-to-poor prognostic significance if present in combination with other genomic changes. Well-established, high-risk prognostic factors in MM include the translocations t(4;14), t(14;16), or t(14;20), the deletion of 17p, and the gain of the 1q arm [87,510,511,512,513,514,515,516].

Chromosomal aberrations involving chromosome 1 are among the most common cytogenetic alterations in MM and include deletions of 1p and amplification of 1q. Several key genes connected to MM etiology are located in the regions affected by some of these aberrations and are often subject to biallelic loss. Thus, tumors in ~4% of MM patients are characterized by a biallelic loss of *FAM46C* resulting from homozygous 1p12 deletions, point mutational inactivation of the *FAM46C*, or both [86,407]. The biallelic loss of *FAM46C* was linked to a shorter overall survival through activation of the PI3K-Akt signaling pathway, which augments MM cell growth and survival [407,517,518]. The *CDKN2C* gene located at 1p32.3 is found deleted in 9–33% of MM tumors that are associated with worse overall survival in MM patients who received ASCT [86,161,407,476,519]. Biallelic inactivation of *CDKNK2C* is frequently observed in MM and is associated with disease progression and has a poor prognosis [407,476].

A heterogeneous class of 1q gain/amplification events is detected in about 40% of patients at the time of diagnosis, and it is one of the most frequent events in MM associated with poor prognosis [520,521,522]. Gain of the 1q may be a result of trisomy or a whole-arm/partial translocations of 1q to the same chromosome or another chromosome. Translocations of the 1q arm may involve several recipient chromosomes, for instance, chromosomes 5, 8, 12, 14, 15, 16, 17, 19, 21, and 22, and most often occur within their telomeric and pericentromeric regions [523,524]. Translocations involving several recipient chromosomes are called “jumping”. The breakpoints on donor chromosome in the case of MM are often localized to the pericentromeric region of the long arm of chromosome 1, with a remarkably high frequency in the regions 1q10–1q11 and 1q12 [525,526,527,528]. Jumping translocations involving 1q are hallmarks of some hematological and other malignancies and can involve interstitial telomeric sequences [524,529] (see Section 6). Segmental copy number variations are also a characteristic of 1q abnormalities in MM and are most often represented by tandem duplications of the 1q21 band. The proportion of these abnormalities and their copy number increases as the disease progresses toward its advanced stages associated with a poor prognosis [512,530,531]. It should be noted that it is the 1q21 amplification that has been classified as the most prognostically significant in the heterogeneous class of 1q abnormalities [530]. The 1q21 region contains several genes whose deregulation or amplification are thought to impact MM progression, including the top candidate, *CKS1B,* encoding the cyclin-dependent kinase regulatory subunit 1B [532] as well as several other genes: *PSMD4* encoding the proteasome 26S subunit, *IL6R* encoding the interleukin 6 receptor, *MCL1* encoding the BCL2 family anti-apoptotic protein, *ANP32E* encoding H2A.Z histone chaperone, *ADAR1* encoding adenosine deaminase, and *ILF2* encoding the interleukin enhancer-binding factor 2 [476,530,531,533]. The 1q21 gains are associated with resistance to bortezomib with no impact on the thalidomide-based regimen [534].

Detailed analysis of 13q deletions revealed several types of aberrations, including small interstitial deletions and gross chromosomal aberrations of the entire 13q. The 13q aberrations were detected in plasma cells of 21–45% of patients with MGUS, and their proportion increased up to 86% for MM and its advanced stages [509,535,536]. Deletion hotspots were at 13q14 where 13q14.11–q14.3 encompasses the minimal deleted region carrying dozens of genes, including the known tumor suppressor *RB1* gene [476,536]. Similar to several other genes discussed above, biallelic inactivation of *RB1* is frequently observed in MM. A complete defect of *RB1* is associated with relapse of the disease and portends poor prognosis [536,537]. Another important region on the 13q is the 13q21.33 band, where *DIS3* is localized. *DIS3* inactivation is frequently associated with 13q deletion. Interestingly, *DIS3* shows the highest frequency of biallelic inactivation among all significantly mutated genes in MM [86,476]. It should be mentioned that 13q abnormalities often coincide with other genomic rearrangements such as t(4;14); thus, their prognostic value is challenging [78]. For instance, *DIS3* mutations are associated with *IGH* translocations t(4;14) and t(14;16) and are less frequently observed in hyperdiploid tumors [28,86,328].

Deletion of 17p results in a loss of the tumor suppressor gene *TP53* located in 17p13, leading to its hemizygotization [476,538]. The occurrence of del(17p) is about 9.5–11% in newly diagnosed MM and increases up to 75% as the disease progresses to its later stages [402,456,513]. Mutations frequently inactivate the remaining copy of the *TP53* in del(17p) MM tumors as the disease progresses, conferring a total defect [29,86,537,538,539]. The *TP53*/17p13 abnormalities resulting in biallelic inactivation of the *TP53* have a clear negative impact on progression-free survival and overall survival, and, thus, are regarded as driver events for MM relapse [61,82,86,328,530,538,539]. The hemizygous 17p deletion, if not accompanied by the inactivation of the remaining copy of *TP53,* is less harmful and has been changed to non-prognostic, although a recent study still suggests an association with poor outcomes [515,530]. 

*MYC* translocations are a distinct group common in MM cases (up to 50%) and involve the 8q24 chromosome region. In most cases, these translocations result in *MYC* upregulation. *MYC* is a transcriptional regulator and a well-established oncogene whose deregulation is associated with many different malignancies, including B cell lymphomas [540] (see Section 1.2). *MYC* is one of the most frequently amplified protein-coding genes in cancer. *MYC* is located in the 8q24 band, a hotspot region for chromosomal rearrangements and SNPs associated with different types of cancer and a frequent site of viral integration [541,542,543]. *MYC* translocations are rarely found in MGUS or stable SMM and are mostly considered to be driver events in MGUS/SMM to MM progression [48,51,62,83,544,545,546]. Because of participation in late-progression events, they often associate with adverse prognosis [456,547,548]. Approximately one-third of 8q24 translocations in MM events involve immunoglobulin (IG) loci (*IGH*, *IGK*, *IGL*-immunoglobulin heavy chain, kappa, lambda genes) where *MYC* juxtaposes to powerful IG enhancers (Figure 4). [545,549,550]. In the rest of the cases, *MYC* is juxtaposed to other super-enhancers associated with genes *NSMCE2*, *TXNDC5*, *FAM46C*, *FOXO3*, *IGJ*, and *PRDM1* [547,549,550,551]. In addition, *MYC* is a direct target of IRF4, which is linked to myeloma predisposition [346,552]. *MYC* rearrangements often coincide with other karyotypic abnormalities: t(14;16) or chromoplexy [550,551]. In addition, *IGL-MYC* translocations constituting approximately 10% of all genomic abnormalities found in MM often coincide with the hyperdiploid state [548].

### 5.2. Mechanisms of Genomic Instability at 14q32 and 8q24 Regions

**Figure 5:**
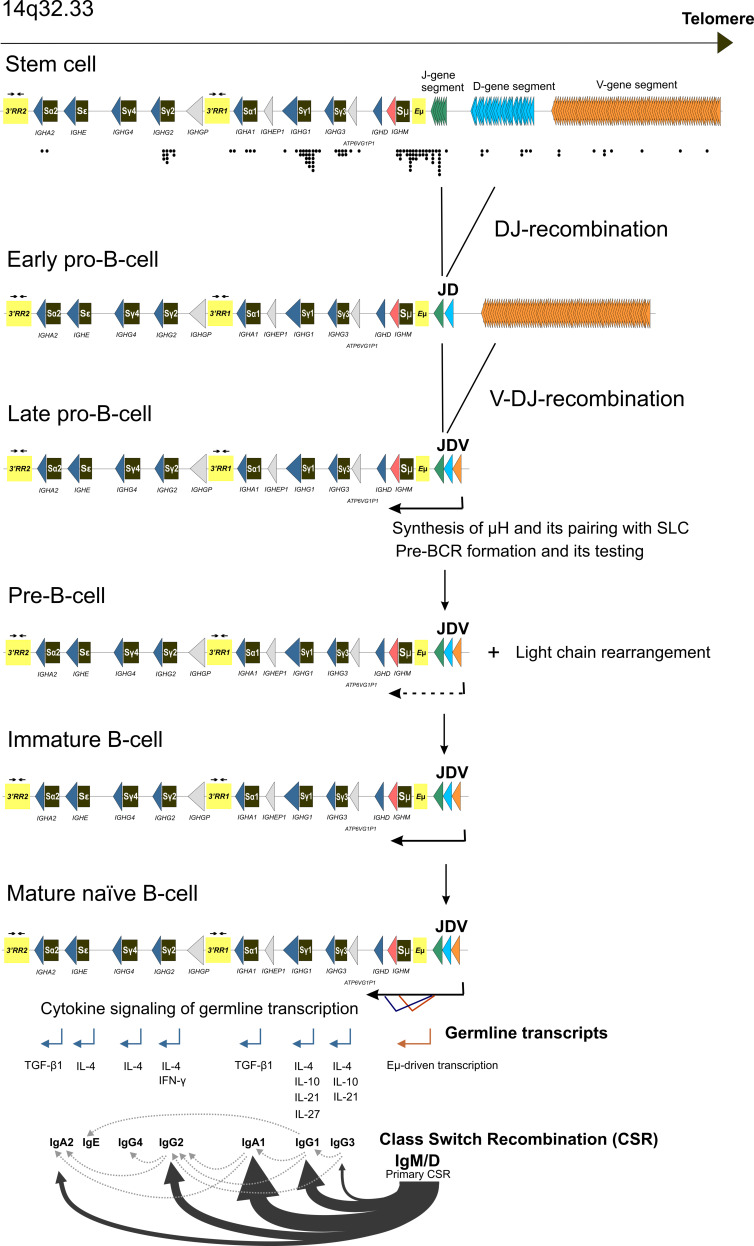
The schematic map of the human *IGH* locus illustrating events occurring during B-cell development. V(D)J rearrangement and CSR are thought to be the two main sources of genomic instability leading to the *IGH* translocations in MM. Translocation breakpoints mapped in MM are shown as black circles under the corresponding elements of the *IGH* locus in the germline configuration on the top. The breakpoints’ coordinates were taken from [553,554]. Then they were located on the chromosome using the GRCh38.p13 reference assembly and were plotted on the map to illustrate their distribution relative to structural elements of the *IGH* locus. Most breakpoints concentrate around Eμ and Sμ as well as in other switch regions such as Sγ1, Sγ2, and Sγ3. Some breakpoints are scattered within V, D, and J regions. CSR depends on the transcription of the switch regions located upstream of most genes encoding the immunoglobulin constant regions. Germline transcription starts from TATA-less promoters located upstream of the small I-exons (not shown) and the switch regions. Transcription goes through the entire S-region and the corresponding C_H_ gene. Isotype switching depends on the corresponding specific transcription (red and blue arrows) and is stimulated by external stimuli such as cytokines (IL-4, IL-21, IL-10, IL-27), CD40 ligands, or T cell-independent signals (e.g., lipopolysaccharide, LPS [555,556,557,558,559,560,561,562,563,564,565,566]). Several studies support the idea of sequential switching of immunoglobulin genes [567,568,569,570]. Primary and secondary switching routes are illustrated as thick, black arrows and dotted, thin arrows, correspondingly. It is generally believed that switch region chromatin structure plays a key role in promoting transcription-coupled AID attack. Changes in histone modifications were observed in switch regions upon cytokine stimulation [571,572,573,574,575,576,577].

The *IGH* locus (14q32.33) is localized at the very extreme terminus of chromosome 14 in the telomeric band. Breakpoints at 14q32.33 initiate a variety of different translocations associated with B cell malignancies and other cancers. Remarkably, some of the partner loci have telomeric (4p16; 6p25) or subtelomeric (16q23) locations [484]. This fact implicates that telomere integrity and interaction between telomeric sequences on different chromosomes might play an essential role in generating these rearrangements. Lymphoid-specific genome rearrangement processes V(D)J recombination and CSR are thought to be the main sources of translocations observed in MM (Figure 5). A proportion of translocation events in MM occurs early in B-cell development at the pro-B cell stage in bone marrow due to aberrant V(D)J recombination [553]. V(D)J recombination is a process leading to diversification of the antigen-binding regions of immunoglobulins and occurs via random recombination between variable (V), diversity (D), and joining (J) genes segments (Figure 5). The other (major) proportion of chromosomal aberrations involving the *IGH* locus in MM occurs in mature B cells during CSR, a process that is responsible for the isotype switching of the heavy immunoglobulin chains. CSR occurs in antigen-activated naïve B-cells soon after infection or immunization (Figure 3) and results in substitution of the constant regions encoding IgM and IgD for the IgG, IgE, or IgA (Figure 5). Both V(D)J recombination and CSR result in intrachromosomal rearrangements, leading to deletion of the genetic information at the *IGH* locus, and are irreversible. In the case of CSR, the sequence encoding the constant C_H_ μ (for IgM) region is substituted for another one encoding for a constant downstream region. The recombination targets during this process are switch (S) regions preceding every constant region except *IGHD* encoding the C_H_ δ (Figure 5). S regions are essential elements providing CSR; they span 2–10 kb and are highly enriched in repeats and G-quadruplex-forming elements necessary for their function. The other elements at the human *IGH* locus are two super-enhancers, the 3′ regulatory regions (3′RRs) and the Eμ intronic enhancer, which are the master regulators of CSR, V(D)J recombination, and SHM and play an essential role in B-cell development.

C_H_ genes in the human genome are arranged into two tandem clusters of genes encoding γ, ε and α isotypes, where Eμ intronic enhancer precedes the *IGHM* gene and 3′RRs are positioned downstream of each cluster (Figure 5) [578,579,580]. Specifically, 3′RRs are located ∼160 kb and ∼275 kb downstream of the Eμ intronic enhancer. Each 3′RRs in humans contains three enhancer elements, hs3, hs1-2, and hs4, which carry binding sites for regulatory and transcription factors [581,582,583]. The 3′RRs are enriched in repetitive sequences, G-quadruplex-forming elements, and carry inverted repeats flanking the hs1-2 regulatory element that makes the structure inside the 3′RR a quasi-palindrome [80,583,584]. The 3′RR and functional S-regions are sufficient to recruit AID and provide CSR when placed at an artificial location in the genome at the *IGK* locus [585]. Defects in the *IGH* regulatory enhancers in mice affect recombination at the *IGH* locus to varying degrees and may lead to a deficiency in antibody production [579,586,587,588].

CSR requires an action of AID and depends on the synthesis of noncoding RNA across the S regions. It was proposed that such transcription would result in R-loop formation and consequent recruitment of AID to the single-stranded regions [564,589,590]. Deamination of cytosine residues generates uracil in the targeted S-region. Subsequent uracil excision from the DNA triggers formation of DNA breaks that are thought to promote recombination. Partner S regions may be located as far as ~270 kb away from each other, so the efficient recombination requires their juxtaposition through long-range chromatin interaction. S/S synaptosome formation is a prerequisite for CSR: Two distant S regions are brought into proximity to facilitate rearrangement. Studies in mice suggest that the *IGH* locus undergoes a remarkable conformational change during the B cell differentiation, including the formation of chromatin loops that provide interaction between distant sequences and play an essential role in the *IGH* locus rearrangements. For instance, chromosome looping allows Vh segments to contact Dh and Jh segments during V(D)J recombination [591]. The contraction of the 2.8 Mb murine *IGH* region depends on WAPL cohesion-release factor, which is controlled by PAX5. Reduced *Wapl* expression causes global alterations in the chromosome architecture [592,593]. Another round of conformational changes occurs before CSR in mature B-cells. The Eμ intron enhancer and the 3′RR get positioned near each other with the formation of the loop [594]. This event is accompanied by a remarkable change in DNA methylation and histone acetylation in the 3′RR [580,595]. Upstream of each acceptor S region is GLT promoter, which, upon B cell activation with cytokines, can be recruited to the Eμ/3′RR. This event enables the effective transcription of the S regions targeted for CSR and their juxtaposition [590,594,596,597]. Cohesin complex and CTCF are implicated in mediating long-range interactions at the *IGH* locus: They tether bases of loops, regulate loop extrusion, and demarcate topologically associating domains [595,598]. The *IGH* locus in both human and murine genomes contain a remarkable number of CTCF sites; some of them are proposed to regulate long-range chromatin interaction, germline transcription, and CSR [598,599,600,601].

**Figure 6:**
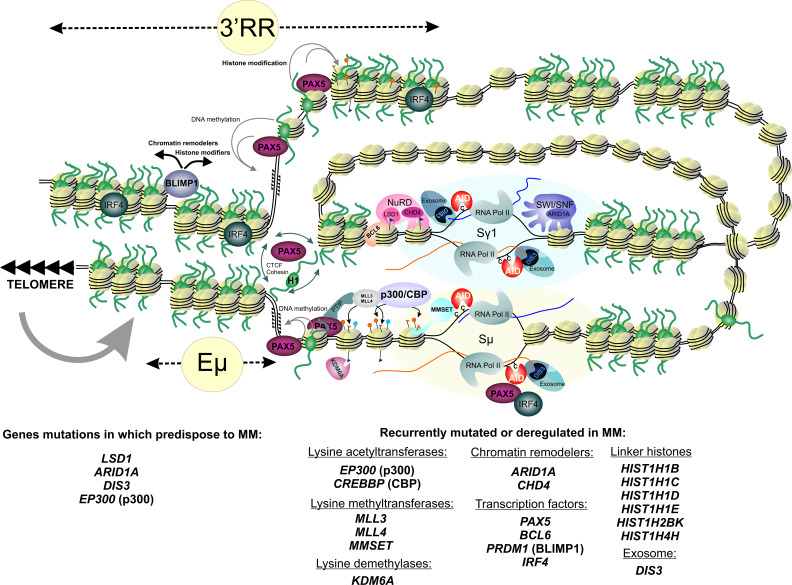
Factors affecting genomic stability and CSR at the *IGH* locus are implicated in MM development. A hypothetical scheme (based on studies in murine and human models) that includes factors essential for the *IGH* locus reorganization and CSR. Many of these factors are encoded by genes mutations in which predispose to MM development and genes that are recurrently mutated in MM. Development of MM may be linked to genomic instability at the *IGH* locus: *IGH* translocations are observed as early as MGUS, are frequent, and predispose to disease development since, in most cases, they fuse various oncogenes to powerful enhancers at the *IGH* locus. Since *IGH* translocations may be linked to aberrant CSR, this is evidence that MM initiation can be simply a result of aberrant CSR. CSR depends on the function of several genetic elements at the *IGH* locus, such as 3’RR, Eμ, and S-regions. It also depends on a number of factors that aid chromatin changes and transcription through S-regions, RNA maturation and processing, and AID recruitment. Transcription factors PAX5 and BCL6 play an essential role in the *IGH* locus contraction and remodeling. PAX5 is a master regulator that binds to the DNA, and recruits PTIP and MLL3/MLL4 methyltransferase complex to specific regions at the *IGH* locus, thus promoting chromatin changes and transcription initiation at S-regions [602,603]. PAX5, together with Linker histone H1 (green spheres with tails), influences DNA methylation and histone modifications at the *IGH* locus [604]. BCL6 binds the promoter region of γ1 GLT and may act by repressing the transcription at S-regions [316]. BLIMP1 and IRF4 bind to 3′RR and regulate transcription at *IGH* locus; BLIMP1 also represses transcription of *AICDA* and *BCL6*, whereas IRF4 activates *AICDA* expression [200,217,605]. AID co-localizes with several transcription factors, including PAX5 and IRF4, at the *IGH* locus, which might play a role in AID targeting to specific locations [606,607]. Chromatin-remodeling factors ARID1A and CHD4 facilitate nucleosome reorganization required for transcription through S-regions. ARID1A is a component of the SWI/SNF complex known to be involved in antisense transcription at the *IGH* locus [608,609]. CHD4 is a component of the NuRD complex, which binds to H3K9me3, an epigenetic mark present at the *IGH* locus during CSR. CHD4 is required for CSR and coimmunoprecipitates with AID in B-cells, and this interaction was proposed to aid AID to target S-regions [610]. LSD1 is a lysine-specific demethylase that associates with the NuRD complex and also interacts with BCL6 [315]. p300 and CBP are closely related acetyltransferases that play a role in transcription activation and enhancer regulation [611,612]. KDM6A acts in concert with MLL3/MLL4 and p300/CBP in the mediation of transcription initiation. MMSET is lysine methyltransferase, which is required for GLT transcription at S-regions [613]. In addition, MMSET promotes AID-mediated DNA breaks at the donor switch region [502]. DIS3 is a component of the exosome, the complex that facilitates 3′-5′ RNA processing and aids in AID recruitment to both DNA strands [331,332,614]. In addition to these mechanisms, the telomeric location of the *IGH* locus suggests that any events that affect telomeric length and structure or telomeric chromatin may influence the CSR and provoke chromosomal aberrations.

Intriguingly, functions of many genes recurrently mutated or deregulated in MM or associated with MM development can be linked to the regulation of the *IGH* locus transcription, V(D)J recombination, and CSR. Therefore, the processes at the *IGH* locus during B cell development can be the key to unraveling the origins of MM (Figure 6).

PAX5 is a major transcription regulator involved in B-cell development, and its mutational inactivation or deregulation is associated with B-cell malignancies [615,616,617]. Mutations in the cis-regulatory elements affecting *PAX5* expression and structural rearrangements involving *PAX5* were identified in MM [60,398,618,619]. Interestingly, *PAX5* regulatory regions, as well regulatory regions of some other B-cell specific oncogenes, get in close proximity and undergo antisense transcription and are targeted by AID, which may explain translocations between these loci and the *IGH* locus and mutagenesis in these regions [614,620]. Differences in *PAX5* expression profiles in B-cells between healthy people and MM patients and unique PAX5 isoforms have been noted in MM [621]. PAX5 has multiple sites of binding at the *IGH* locus and participates in spatial locus contraction necessary for its rearrangement and V-DJ recombination [622,623,624,625,626]. The 3′RRs in mice are interspersed with multiple PAX5 binding sites and undergo dynamic changes in PAX5-binding during CSR [596]. PAX5, together with Linker histone H1 is involved in B-cell specific DNA methylation and histone modifications at the 3’RR in mice [604]. DNA is hypermethylated at B-cell specific intronic enhancer regions in MM but not in normal plasma cells, and these sites overlap with binding sites of some transcription factors, including PAX5 [428]. PAX5 binding to the DNA recruits PTIP and promotes binding of MLL3/MLL4 histone lysine methyltransferase complex to specific regions at the *IGH* locus in mice, which promotes chromatin changes and transcription initiation at switch-regions critical for CSR [602,603]. MLL3/4 containing COMPASS complex includes PTIP and KDM6A and functions with p300/CBP in the *de novo* “commissioning” of enhancers [611]. As was discussed in Section 3, mutations in *MLL3*, *MLL4*, *KDM6A*, *EP300*, *CBP*, and, to a lesser extent, *PTIP* were all found in MM.

Interestingly, the *IGH* locus is one of the most heavily transcribed in plasma cells. Antisense transcription has been observed from variable region exons and from S regions and is thought to be implicated in AID-targeting [608,614]. SWI/SNF chromatin remodeling complexes are recruited to *IGH* locus and facilitate antisense *IGH* transcription and accessibility of *IGH* for recombination in B cell precursors [609]. As was discussed earlier (Section 2 and Section 3), genes encoding SWI/SNF complexes are altered in many cancers, including MM, and mutations in the genes encoding components of SWI/SNF complex are linked to MM predisposition. In hematopoietic progenitors and non-B lineage cells, *IGH* is localized to the nuclear periphery [627]. More specifically, in fibroblasts, the *IGH* locus was shown to be tethered to the nuclear lamina, where its transcription is repressed [628]. Relocalization of the *IGH* locus from the periphery to the nuclear interior occurs in pro-B cells that, along with intergenic and antisense transcription and action of chromatin remodelers, facilitates genes shuffling during V(D)J recombination and further maturation of the antigenic repertoire [627,629,630]. It has been proposed that in cells, there is a special perinucleolar recombination compartment where the *IGH* locus undergoes rearrangements during B-cell maturation and where the activity of AID and RAG1/2 can be sequestered [631].

AID recruitment to the transcribed S-regions at the *IGH* locus is aided by the exosome. Exosome interacts with the RNA Polymerase II and may facilitate exonucleolytic degradation of a 3′ free RNA end on backtracked complexes, thus helping to generate ssDNA substrate for AID. The *DIS3* gene that we discussed in Section 2 and Section 3 is a catalytic component of the exosome and is implicated in regulating of AID-dependent mutagenesis and recombination at the *IGH* locus [331,332]. Other genes involved in transcription regulation, RNA processing, and maturation might also impact the complex process of the *IGH* locus reorganization. More research is required to understand the interrelation between various processes at the *IGH* locus and MM development.

Similar to *IGH* translocations, some types of *MYC* translocations are thought to originate from the aberrant, B-cell specific processes: V(D)J recombination, *IGH* switch recombination, and SHM [632]. Studies in a murine model with AID-deficient mice have suggested that AID plays a crucial role in *MYC/IGH* translocations [633]. However, the localization of *MYC* translocations breakpoints in the *IGH* locus in newly diagnosed MM patients showed that they are spread out over the constant regions with no apparent association with AID motif clusters, pointing to a different mechanism of their generation [551]. 8q24 is a very peculiar region – it is a so-called “gene desert” apparently representing an important source of genomic instability and translocation events. This region is almost devoid of protein-coding genes but contains multiple regulatory elements, and it is a source of noncoding DNA transcription [542]. Remarkably, the breakpoints at 8q24 found in MM surround a region of active chromatin characterized by H3K27Ac, H3K36me3, and H3K4me1 marks, as well as DNaseI hypersensitivity [551]. Human Plasmacytoma Variant Translocation 1 (*PVT1*) gene is located in the same chromosome band 8q24 just 54Kb away from *MYC*, and is frequently involved in structural rearrangements observed in MM, primarily in *MYC* rearrangements [634]. *PVT1* encodes 52 noncoding RNAs, including circular ncRNAs, linear ncRNAs, and microRNAs functioning as negative regulators of immune response and associating with hematological malignancies and other types of cancer [635]. 8q24 translocations result in overexpression of *MYC* as well as *PVT1* [551]. Besides that, the *PVT1* promoter can inhibit *MYC* expression by outcompeting intragenic enhancers located in the *PVT1* locus [636]. Interestingly, in mice, *MYC* is recruited to the same transcription factory as *IGH*, which might facilitate spatial closure of these regions and consequently specific chromosomal translocations involving *IGH* and *MYC* [637]. *MYC* and *IGH* loci in mice have been shown to interact physically with each other and are tethered to the nucleolus via the nucleolar organizing regions at their chromosomes [638,639]. Colocalization of *IGH*, *MYC*, and *CCND1* loci at a time when B cells undergo V(D)J recombination or SHM/CSR might favor certain types of translocations [640,641].

### 5.3. Complex Chromosomal Rearrangements in MM and Their Mechanisms

Several types of complex chromosomal rearrangements have been observed in MM. They include chromoplexy, templated insertions, and chromothripsis [61,83,619,642,643,644]. Chromoplexy represents a complex genomic alteration leading to balanced rearrangements (i.e., without apparent cytogenetically detectable loss of genetic material) at multiple breakpoints of several chromosomes that are believed to occur in a coordinated fashion [645,646]. In chromoplexy, the rearrangements often occur as nearly precise junctions without large deletions; however, small deletions may occur involving those affecting function of oncosuppressors [647]. NHEJ is thought to be a primary mechanism of the generation of such rearrangements. Chromoplexy is considered a late-progression event in MM, possibly linked to positive selection and drug resistance development [61].

Templated insertions are a distinct class of structural rearrangements characterized by DNA segments copied from different areas of the genome, joined and inserted as one contiguous sequence into a single derivative chromosome [645,648]. These insertions are found in about 20% of MM cases and are clonal events, suggesting their early origin in MM development [61,83,619]. Templated insertions in MM can occur in one or several steps and lead to amplification of oncogenes (e.g., *MYC*) or their juxtaposition to powerful enhancers [61,547,619,645]. The term “chromoanasynthesis” is often used in literature to describe a replication-based process leading to complex rearrangements that are templated and characterized by gain or amplification of chromosomal segments [647]. Break-induced replication (BIR) is a specific HR mechanism responsible for the repair of DNA strand breaks independently of each other that involves template switching and replication, and that can account for complex rearrangements seen in cancers [649,650]. BIR can involve multiple template switching events between dispersed repeated sequences [651]. The two related processes termed microhomology-mediated, break-induced replication (MM-BIR) and fork-stalling and template switching (FoSTeS) are discussed to underlie complex genomic rearrangements that resemble the pattern of templated insertions observed in cancer cells [652,653,654,655].

Chromothripsis is a massive ‘‘catastrophic’’ structural rearrangement of the genome that involves the shattering of one or several chromosomes to pieces followed by their reassembly in a new order with the formation of new chromosomes. Chromothripsis is believed to occur in one step and, besides gene order changes, can result in deletions or amplification of certain regions. Chromothripsis also causes extrachromosomal structures, termed double minutes, containing pieces of shattered chromosomes with altered chromatin structure. Initially, chromothripsis was thought to be a rare event in cancer progression, but recent studies reveal that it is not. Chromothripsis occurs with a frequency of up to 30% in osteosarcoma and glioblastoma, about 60% in melanoma, and up to 36% in MM [61,619,647,648,656]. Chromothripsis, as well as templated insertions, are currently viewed as relatively early events in MM development that can drive tumor development [61,62]. Chromothripsis is associated with known high-risk genetic alterations in MM, including *IGH* translocations involving *MMSET*, *MAF*, or *MAFB*; biallelic inactivation of *TP53*, deletion of 1p12, and high APOBEC mutational burden [619]. In addition to that, chromothripsis was associated with poor outcomes and was linked to treatment in MM [619,642,644,657]. For instance, chromothripsis occurs more often in patients with bortezomib-resistant MM that can be interpreted as a drug-induced response [657].

Several mechanisms have been proposed to explain the nature of chromothripsis. The initiating events are thought to be telomeres’ attrition and dysfunction leading to telomere-telomere fusions and consequent breakage-fusion-bridge (BFB) cycles [656,658,659,660,661]. The dicentric chromosomes formed during this process are subject to processing aimed at their isolation or resolution during cell division. In one scenario, the dicentric chromosomes or chromosomes’ fragments can be compartmentalized into micronuclei. Such chromosomes become physically isolated from the rest of the genome in a microenvironment where they can undergo abnormal repair processes resulting in chromosomes’ shattering [662,663,664,665]. Micronuclei are often found in cancer cells and are characterized by abnormalities in DNA replication, transcription, and nuclear envelope structure [666]. Formation of micronuclei and chromosome pulverization distal to 1q12 have also been described in MM patients with aberrations involving 1q [526,644]. Another model proposes that chromatin bridges formed during cytokinesis are pulverized via concerted action of TREX1 exonuclease and APOBEC3A/B cytidine deaminases [658,667,668]. TREX1 generates ssDNA that is deaminated by APOBEC3A/B enzymes with the consequent repair of abasic sites leading to DNA breaks. This mechanism implies that chromothripsis and *kataegis* events can be interrelated. In some cancers, chromothripsis and other structural variations indeed associate with *kataegis* [452,648,669,670]. Remarkably, TREX1 is a cytoplasmic nuclease requiring nuclear envelope rupture to access the chromosomal DNA [658]. Other models suggest that multi-invasion-induced rearrangements (MIR) and MM-BIR can contribute to complex structural rearrangements observed in chromothripsis [668,671].

## 6. Telomere Maintenance Pathways and MM Risk

Multiple lines of evidence suggest that a major risk of developing MM can be linked to defects in telomere maintenance mechanism leading to changes in telomere length and structure. Telomere shortening has been observed in patients with plasma cell disorders [672]. MM tumor cells can maintain stable short telomeres and telomere length is a prognostic marker in MM [673,674]. Telomerase activity negatively correlates with MM survival rate and telomere length in MM, according to telomerase maintaining of short and critically short telomeres [675,676,677]. At the same time, a small proportion of MM patients have very long telomeres alluding to Alternative Lengthening of Telomeres (ALT) in the progress of the disease.

A strong correlation was found between telomerase activity and 1q jumping translocations [675], implicating telomerase-dependent DSB repair or targeted telomere insertion in the process of 1q rearrangements [529,678,679,680]. It is noteworthy that a block of Interstitial Telomeric Sequences (ITSs) was reported at 1q12 [681]. ITSs are important hotspots of DSBs and recombination and have been implicated in the formation of jumping translocations [529,682,683,684]. ITSs can also impact 3D nuclear architecture through interaction with telomeres and ITL loops mediated by TRF2 and Lamin A/C [685,686]. Changes in the 3D nuclear architecture accompanied by telomere attrition and formation of telomere aggregates have been noted during disease progression from MGUS to MM [687]. A number of telomeric signals were reported to be significantly elevated in MM compared to MGUS, indicating either addition of new chromosomes or amplification of interstitial telomeric repeats [688]. Other than telomerase activation, a significant increase of *TRF2* and *TANK1* expression levels in patients with MGUS and MM and *TRF1* in patients with MM was observed [676]. Telomere dysfunction and attrition can promote recombination between telomeres, followed by telomere-telomere fusions and BFB cycles and eventually chromothripsis, as was discussed in Section 5.3.

Several SNPs potentially affecting genes implicated in telomere length regulation have been associated with the increased susceptibility to MM (Table S3). Some of these polymorphisms lie within regions occupied by *TERC* and *TERT* genes encoding for telomerase RNA and telomerase catalytic subunit, correspondingly. These SNPs may affect telomerase activity [343,689]. The risk of B-cell lymphomas, including MM, is associated with longer telomeres [690,691]. For instance, a common [C] allele of rs10936599 is strongly associated with increased telomere length and susceptibility to MM and colorectal cancer [343,689,691,692,693,694]. Although the association of this SNP with telomerase activity remains controversial, the carrier status of [C] allele for the rs10936599 was associated with increased expression of *TERC* in another tumor of differentiated B-cells, Chronic Lymphocytic Leukemia (CLL), and susceptibility to CLL [695]. Carriers of another allele [T] at rs10936599 demonstrated shorter telomeres compared to homozygous [C] carriers [696]. rs10936599 has a genetic association with SNP rs2293607, which maps 63bps 5′ to *TERC* and impacts *TERC* mRNA expression [343,692]. The rs2293607 [A] allele results in longer telomeres in colorectal cancer cell lines [692]. Another SNP, rs2242652, which lies in intron four of the *TERT* gene, is associated with a decreased risk of MM [689]. The minor allele [A] of this SNP downregulates *TERT* promoter activity and has been associated with the increased breast and ovarian cancer but decreased risk of prostate cancer and MM [689,697]. Additionally, SNP rs10936600 is located in the same 3q26.2 locus and is associated with an increased risk of MM [354]. This SNP is located within the *LRRC34* gene—a predicted ribonuclease inhibitor. Interestingly, the telomere-associated SNP rs10936599 affected *LRRC34* expression, and the risk allele for shorter telomere length [T] was associated with reduced *LRRC34* expression [698]. Another SNP, rs58618031, associated with increased risk of MM, is located in the band 7q31.33, which lies within the *POT1-AS1* lncRNA gene near the *POT1* gene and might affect *POT1* expression [345]. POT1 is a component of the Shelterin complex protecting telomeres by interaction with the single-stranded G-rich tip of a telomeric DNA [699]. *POT1* mutations are commonly observed in some B-cell malignancies such as CLL [700]. Increased *POT1* expression was linked to the transformation from MGUS to MM [701]. In addition to that, differences in the expression of other telomere maintenance genes between MGUS and MM were reported [673,676]. *RECQL*, encoding RECQ1 helicase associating with telomeres in ALT cells [702] is significantly overexpressed in MM, which correlates with resistance to melphalan and bortezomib [703,704]. *RECQL* overexpression in MM is linked to aberrant methylation of miR-203 [703].

Genes *LSD1/KDM1A*, *ARID1A*, *USP45*, *DIS3*, and *EP300*, where family risk mutations for MM have been reported (see Section 2), are also linked to the telomere maintenance pathway [305,319,330]. LSD1 interacts with telomeric repeat containing RNA TERRA and regulates silencing at telomeres and telomere length [314,705,706]. ARID1A represses *TERT* transcription and promotes telomere cohesion and protection [489,707,708]. USP45 controls the activity of the ERCC1-XPF complex that interacts with Shelterin component TRF2 and facilitates nucleolytic processing of the 3′-end at uncapped telomeres, promotes t-loop formation, and plays a role in telomere integrity [325,709]. DIS3 is implicated in the processing of a variety of RNA transcripts, including telomerase RNA (hTR) precursors, which can affect telomerase activity [710]. As was discussed above, RNA exosome and DIS3 have been implicated in mutagenesis and recombination in the *IGH* locus as well, specifically in AID targeting during CSR [331,332,711]. DIS3 can potentially play a dual role in MM, affecting both telomerase activity and recombination at the *IGH* locus, where primary structural aberrations during the development of MM occur. p300 acetylates TRF2, and this modification stabilizes the TRF2 protein by inhibiting its ubiquitin-dependent proteolysis and promotes efficient binding of TRF2 to telomeres [712]. p300 also associates with telomeres and catalyzes acetylation of H3K27 [713]. It is necessary to re-emphasize, that the position of the *IGH* locus itself in the telomeric region could be an important factor influencing its chromatin structure, 3D position in the nucleus, and the DNA repair that can ultimately forge structural aberrations at the *IGH* locus. In conclusion, telomere maintenance pathways play a significant and multifaceted role in the development of MM. Telomerase might be the general target of the PIs, such as bortezomib, carfilzomib, epoxomicin, and others, affecting its activity both transcriptionally and post-translationally [714,715].

## 7. Conclusions and Further Perspectives

We have reviewed the current understanding of the genetic heterogeneity and variability of MM and discussed different aspects of this phenomenon: types of genetic alterations found in MM, the molecular mechanisms of MM genome plasticity, as well as the dynamics of genetic changes during the development of the disease and its treatment. Owing to extensive research of MM and the wide use of NGS, the molecular mechanisms of MM development are much better understood now. Dozens of recurrently mutated genes were found in MM genomes. NGS also contributed to a better understanding of predisposition to MM and helped in a precise analysis of structural genome variations seen in MM. Recent WES studies revealed genomic signatures that may be useful in molecular individualizing of B-cell neoplasms and a better understanding their origin. For instance, the mutational landscapes of MM, plasmablastic lymphoma (PBL), and diffuse large B-cell lymphoma (DLBCL) differ in the proportion of mutations affecting the signaling pathways RAS/MAPK, JAK/STAT, and NOTCH. Another remarkable feature is different frequencies of mutations affecting *DIS3* and *TET2* genes [716,717,718].

Over the past few years, the overall survival of patients with MM has been significantly increased. This improvement is made possible by the development of new drugs and patients’ stratification based on genetic factors. Despite this undoubted success, the disease remains incurable and relapses in most patients after a certain period. Moreover, some patients do not ever respond to any currently available therapy. Several questions have to be answered. It is vital to find why some patients do not respond to therapies or develop drug resistance. What is the effect of the treatment on tumor evolution? This problem becomes even more important nowadays because new drugs for MM treatment appear every year. Some of these drugs are based on new principles of action (e.g., monoclonal antibodies, CAR T-cell therapy, targeted delivery of chemotherapeutic drugs, such as melphalan flufenamide or antibody–drug conjugates), while others are developed on a platform of new knowledge about the genetic plasticity of the MM genome (e.g., histone deacetylase inhibitors, drugs targeting DNA or histone’s methylation pathways, pyrimidine nucleoside analogs) [103,130,132,719,720,721]. Next-generation IMiDs, PIs, agents that target the ubiquitin proteasomal cascade, and signaling pathways, are being developed to overcome the limitations of existing therapies [719,722,723].

While genome instability has been acknowledged as the driving force in MM development, we still do not know much about the combined contribution of the intrinsic genetic plasticity of MM and therapy to the progression of the disease [724]. Epigenetic factors and non-coding RNome add another level of complexity [725,726].

An urgent problem is a search for new risk factors and more fractional patient stratification based on certain risk factors that will allow using highly personalized regiments. Though the NGS techniques led to progress in understanding pathogenesis and progression of MM, WES and WGS are applied in most cases for basic research but not as a diagnostic technique that would help assess the severity of the condition and response to treatment. The WGS approach has cogent value, but bioinformatics analysis of NGS data for many samples is time-consuming and is not widely used in routine clinical care. The cytogenetic analysis by fluorescence in situ hybridization (FISH), karyotyping, or single nucleotide polymorphism arrays are prevalent approaches in current clinical practice for MM analysis. Custom target NGS has significant advantages to identify mutations, copy number alterations, and translocations in clinical practice [328,554,727,728]. The ultra-low pass whole-genome sequencing (ULP-WGS) approach can be an alternative method to WGS for the detection of copy number aberrations [48,437,439,729]. The DNA sequence data in combination with the whole transcriptome approach (RNA-Seq) helps to find driver mutations, molecular heterogeneity in different stages of MM, assess drugs response, and guide therapeutic decisions [730,731,732]. Newer studies attempted to create a platform for precision treatment and extend the application of WGS and RNA-Seq approach for MM in clinics [80,730,732], but the low level of standardization of this technique so far precludes its routine use in clinical practice. In the future, MM research utilizing comprehensive genomic data in combination with clinical evidence will robustly identify genetic markers associated with MM and eventually lead to improved diagnostics and treatment of MM.

## Supplementary Materials

The following are available online at https://www.mdpi.com/article/10.3390/cancers13235949/s1. Table S1: NGS-based studies of MM genomes, Table S2: Recurrently mutated genes in MM, Table S3: SNPs associated with MM/MGUS according to GWAS and other studies.
