# Supplementary material for: Genome Instability in Multiple Myeloma: Facts and Factors
Source: Cancers (Basel). 2021 Nov 26;13(23):5949. doi: 10.3390/cancers13235949 (PMC8656811; doi:10.3390/cancers13235949)
Supplement: Supplementary file 1 [file cancers-13-05949-s001.zip › cancers-1321436-supplementary/Tables S1,S2.pdf]

cancers

MDPI

*Supplementary Materials*

# Genome Instability in Multiple Myeloma: Facts and Factors

**Anna Y. Aksenova, Anna S. Zhuk, Artem G. Lada, Irina V. Zotova, Elena I. Stepchenkova, Ivan I. Kostroma, Sergey V. Gritsaev and Youri I. Pavlov**

**Table S1.** NGS-based studies of MM genomes.

| Type | Patients/samples | Experimental design | Description | Reference |
|---|---|---|---|---|
| WGS/WES/single cell RNA-seq/RNA-seq | 14 patients with 29 samples | Tumor-normal pairs, tumor DNA from CD138+ PCs BM and normal DNA from skin biopsy, MM patients at multiple stages of disease with combination of treatment timeline and NGS data types | Investigation of evolution in plasma cell population structure from SMM to MM and further disease progression | [1] |
| WGS | 74 patients with 113 samples, of which 25 samples were newly sequenced and other samples from [2] [3] [4] [5] | Tumor-normal matched pairs, tumor DNA from CD138+ PCs BM and normal DNA from marrow T cells or peripheral blood mononuclear, samples from different stages MGUS, SMM, MM (MGUS – 19, SMM – 14, MM – 80 samples), also serials samples for 11 patients at SMM and MM progression | Study of the relationship between MGUS, SMM and MM | [6] |
| WGS/WES | WGS data for 30 patients with 89 samples and WES data for 973 patients from CoMMpass trial | Tumor-normal matched pairs, tumor DNA from CD138+ PCs BM and normal DNA from peripheral blood mononuclear cells, serial sampling for different time points SMM (11 samples), newly diagnosed MM (15 samples) and relapsed MM (41 samples) | Investigation of mutational signature during MM pathogenesis | [2] |
| WGS/RNA-seq | 211 patients | Tumor-normal unmatched pairs, tumor DNA from CD138+ PCs BM, and unmatched-normal DNA from anonymous donors, Patients with MM | Validation of WGS and RNA-Seq results with gold standard FISH diagnostics in MM patients | [7] |
| WES/deep targeted NGS | 214 patients, WES data for 166 tumor samples and deep targeted sequencing for 48 tumors | Tumor-normal matched pairs for 72 samples and tumor only for 95 samples, patients with SMM | Identification of genetic alterations with higher risk for progression of SMM to MM | [8] |

*Cancers* **2021**, *13*, 5949. https://doi.org/10.3390/cancers13235949 www.mdpi.com/journal/cancers

| | | | | |
|---|---|---|---|---|
| ULP-WGS/targeted NGS | 31 patients | Tumor-normal pairs, tumor DNA from CD138+-purified PCs BM, 6 patients with MGUS and 25 patients with SMM | Applying ultra-low-pass whole genome sequencing (ULP-WGS) and targeted NGS of 56 genes to identify clinically relevant gene mutations and copy number alterations in SMM | [9] |
| WES | 4 patients with 21 samples | Tumor-normal matched pairs, tumor DNA from CD138+-purified PCs BM, normal DNA from skeletal muscles, patients with MM at relapse, tumor sample from a different biopsied body site | Characterization of evolutionary process in MM at the time of aggressive relapse in patients previously exposed to high-dose melphalan | [10] |
| WGS/ sequencing panel | 612 patients with WGS data from CoMMpass trial and 113 patients with target sequencing data | Tumor samples for 612 newly diagnosed myeloma (NDMM) patients and 23 patients with MGUS and 90 patients with SMM | Study the role of secondary genetic events activating MYC, RAS or NFkB pathways in MM progression | [11] |
| ULP-WGS | 20 patients | Tumor-normal matched pairs, tumor DNA from CD138+ purified PCs BM, and normal DNA from peripheral blood, patients with MM | Examination of ULP-WGS as alternative approach for the clinical diagnostics of MM | [12] |
| WGS/WES | WGS data for 30 patients with 67 samples and 804 patients with WES data from CoMMpass trial | Tumor-normal matched pairs, tumor DNA from CD138+ purified PCs BM, and normal DNA from peripheral blood mononuclear cells, serial sampling for different time points smoldering MM, symptomatic and relapsed MM | Study of driver events in MM, identification of SVs and complex events in MM; chromothripsis and cycles of templated insertions are a key drivers of disease initiation, focal deletions on oncogenes and whole-genome duplication acquired during disease progression and could be involved in treatment resistance | [13] |
| WGS/WES | 850 samples with WGS data and WES data on 874 samples from CoMMpass | Tumor-normal matched pairs, newly diagnosed MM patient | Identification of the mutational signatures for MM in data from the CoMMpass | [14] |
| WGS | 795 patients from CoMMpass | Tumor-normal matched pairs, newly diagnosed MM patients | Identification of structural variants in MM | [15] |
| WES | 9 patients with 53 samples | Tumor-normal pairs, tumor DNA from CD138+ PCs BM, serial samples for SMM patients were analyzed | Study the genetic variations underlying myeloma initiation and progression SMM to MM | [16] |
| WES | 1157 public available samples from CoMMpass and [3,17] | Tumor-normal pairs, MGUS/SMM and MM patients | Study APOBEC activity of cytidine deaminases in MM progression; global overview on the contribution of mutational processes in different phases from MGUS and SMM to MM | [18] |

| | | | | |
|---|---|---|---|---|
| WGS/WES | 765 patients with WGS data and 804 patients WES data from CoMMpass | Tumor-normal matched pairs, tumor DNA from CD138+ PCs BM, MM patients | Identify novel coding and non-coding drivers in MM development | [19] |
| WES | 1273 samples from Myeloma Genome Project | Tumor-normal matched pairs, newly diagnosed patients with MM | Identify driver genes alterations for MM | [20] |
| WGS | 11 patients with 21 samples | Tumor-normal matched pairs, tumor DNA from CD138+ PCs BM and normal DNA from peripheral blood mononuclear cells, samples from SMM patients progressing to MM | Identify mutations and structural rearrangements at SMM | [5] |
| WES /RNA-seq | 64 patients | Tumor-normal matched pairs, tumor DNA from CD138+ PCs BM and normal DNA from peripheral blood granulocytes relapsed and/or refractory MM patients | Creating a novel computational platform for precision medicine of relapsed and/or refractory MM on of DNA and RNA sequencing data | [21] |
| WES/RNA-seq | 718 patients from CoMMpass | Tumor-normal matched pairs, MM patients | Compare two cohorts of Caucasians and African American MM patients to identify mutation frequencies in MM genome across populations | [22] |
| WES/ ULP-WGS/ deep targeted NGS | 186 samples, WES data for 70 samples, ULP-WGS and targeted deep sequencing for 116 samples | Tumor-normal matched pairs. SMM patients | Study specific genomic alterations are associated with high-risk SMM and low-risk SMM to distinguish progressors from non-progressors in SMM | [23] |
| WGS | 11 patients with 22 samples | Tumor-normal matched pairs, tumor DNA from CD138+PCs BM, patients SMM/MGUS stage at the time of progression to symptomatic MM | Study contribution of AID in mutational processes on early stages of MM | [24] |
| WES | 463 patients | Tumor-normal matched pairs, tumor DNA from CD138+PCs BM and normal DNA from peripheral blood, MM patients on different treatment from clinical trial NCRI Myeloma XI (NCT01554852) | Identify significantly mutated genes and clinically relevant mutations in MM | [25] |
| WES | 67 patients with 84 samples | Tumor-normal matched pairs, tumor DNA from CD138+ PCs BM, and normal DNA originated from peripheral blood mononuclear cells, MM patient with serial sampling for different time points: disease progression or relapse post treatment and relapse/progression after further treatment | Identify new candidate genes, including truncations of *SP140*, *LTB*, *ROBO1* and clustered missense mutations in *EGR1* | [17] |

| | | | | |
|---|---|---|---|---|
| WES | 10 patients | Tumor-normal matched pairs, tumor DNA from CD138+ PCs BM, patients with asymptomatic monoclonal gammopathies that preceded MM | Comparison of serial exomes in progressive versus non-progressive precursor states in MM | [26] |
| WGS/WES | 203 patients, 177 patiens with WES data and 26 patients with WGS data | Tumor-normal matched pairs, tumor from bone marrow aspirate and normal from blood, patients with MM | Identify significantly mutated genes and copy number alterations mutations in *KRAS*, *NRAS*, *BRAF*, *FAM46C*, *TP53*, and *DIS3* | [3] |
| WGS/WES | 38 patients, 23 patients with WGS and 16 patients with WES | Tumor-normal matched pairs, tumor from bone marrow aspirate and normal from blood patients with MM | Identify coding and non-coding mutations involved into the pathogenesis of MM | [4] |

PCs BM plasma cells from bone marrow aspirates.

**Table S2.** Recurrently mutated genes in MM.

| Pathway and Mechanism | Gene | Description | Impact on survival | % of patients with the mutation (Reference) |
|---|---|---|---|---|
| **RAS/MAPK-pathway** Activation of MAPK signaling cascade reduces apoptosis and promotes malignant plasma cell proliferation | *KRAS* | Kirsten rat sarcoma viral oncogene homolog, GTPase Association with t(11;14) [25]. 73% KRAS mutations are clonal, 27% are subclonal [3] | No [25] | 50 [4] |
| | | | | 23 [3] |
| | | | | 48 [17] |
| | | | | 21.2 [25] |
| | | | | 21.84 [20] |
| | | | | 24 [19] |
| | | | | 19.8 [27] |
| | *NRAS* | Neuroblastoma *RAS* viral oncogene homolog. GTPase | No [25] | 50 [4] |
| | | | | 20 [3] |
| | | | | 17.44 [20] |
| | | | | 19.4 [25] |
| | | | | 26 [27] |
| | *BRAF* | B-Raf proto-oncogene, serine/threonine kinase | No [25] | 4 [4] |
| | | | | 15 [17] |
| | | | | 6 [3] |
| | | | | 6.7 [25] |
| | | | | 7 [19] |
| | | | | 8.01 [20] |
| | *DUSP2* | Dual specificity protein phosphatase 2, a negative regulator of MAPK/ERK, SAPK/JNK and p38 | (ND) | 3.6 [20] |

| | | | | |
|---|---|---|---|---|
| | *FGFR3* | Fibroblast growth factor receptor 3 Only mutated in the t(4;14) group [25] | ND | 2.16 [25] |
| | | | | 3 [19] |
| | | | | 3.93 [20] |
| | *PTPN11* | Protein tyrosine phosphatase, non-receptor type 11 | ND | 2 [19] |
| | | | | 2.28 [20] |
| | *RASA2* | RAS P21 protein activator | ND | 1 [19] |
| | | | | 1.26 [20] |
| | *NF1* | Neurofibromatosis type 1 protein | ND | 2.44 [20] |
| | *ROS1* | Proto-oncogene tyrosine-protein kinase | ND | 1.73 [25] |
| | *RET* | Proto-oncogene tyrosine-protein kinase receptor | ND | 1.3 [25] |
| | *ERBB4* | Receptor tyrosine-protein kinase | ND | 1.08 [25] |
| | *ERBB2* | Erb-B2 receptor tyrosine kinase 2 | ND | 0.65 [25] |
| | *FLT3* | Fms-like tyrosine kinase 3 | ND | 1.08 [25] |
| | *FGFR2* | Fibroblast growth factor receptor 2 Tyrosine-protein kinase | ND | 0.65 [25] |
| | *FGFR4* | Fibroblast growth factor receptor 4 Tyrosine-protein kinase | ND | 0.22 [25] |
| | *KIT* | KIT Proto-oncogene, receptor tyrosine kinase | ND | 0.65 [25] |
| | *MAP2K1* | Dual specificity mitogen-activated protein kinase kinase 1 | ND | 0.65 [25] |
| | *EGFR* | Epidermal growth factor receptor | ND | 0.43 [25] |
| | *EPHB2* | Ephrin type-B receptor 2 | ND | 0.43 [25] |
| | *PDGFRA* | Platelet-derived growth factor receptor alpha | ND | 0.43 [25] |
| | *DDR2* | Discoidin domain-containing receptor 2 | ND | 0.22 [25] |
| | *MET* | MET proto-oncogene, receptor tyrosine kinase | ND | 0.22 [25] |
| **NF-kB pathway** Activation of the NF-kB pathway leads to alterations in apoptosis, differentiation and proliferation, immunity, inflammation, lymphopoiesis | *TRAF3* | TNF receptor associated factor 3 | ND | 2.6 [4] |
| | | | | 2.6 indel [4] |
| | | | | 5 [3] |
| | | | | 3.7 [25] |
| | | | | 7 [19] |
| | | | | 5.26 [20] |
| | *TRAF2* | TNF receptor associated factor 2 | ND | 2.36 [20] |
| | | | | 2 [19] |
| | *CYLD* | Cylindromatosis (Turban tumor syndrome) lysine 63 deubiquitinase | ND | 2.6 indel [4] |
| | | | | 2 [3] |
| | | | | 2.4 [25] |
| | | | | 2 [19] |

| | | | | |
|---|---|---|---|---|
| | | | | 3.38 [20] |
| | *LTB* | Lymphotoxin beta<br>TNFactor ligand superfamily member 3 | ND | 3 [25] |
| | | | | 3 [19] |
| | *BTRC* | Beta-transducin repeat containing E3 ubiquitin protein ligase | ND | 2.6 [4] |
| | *CARD11* | Caspase recruitment domain family member 11 | ND | 5.3 [4] |
| | *IKBIP* | IKBKB interacting protein | ND | 2.6 [4] |
| | *IKBKB* | Inhibitor of nuclear factor kappa B kinase subunit beta | ND | 2.6 [4] |
| | *MAP3K1* | Mitogen-activated protein kinase kinase kinase 1 | ND | 2.6 translocation [4] |
| | *MAP3K14* | Mitogen-activated protein kinase kinase kinase 14 | ND | 2.6 indel [4] |
| | *RIPK4* | Receptor interacting serine/threonine kinase 4 | ND | 2.6 [4] |
| | *TLR4* | Toll like receptor 4 | ND | 2.6 [4] |
| | *TNFRSF1A* | Tumor necrosis factor receptor superfamily member 1A | ND | 2.6 [4] |
| | *PRKD2* | Serine/threonine-protein kinase D2 Correlated to t(4;14) [25]. | ND | 2.1 [25] |
| | | | | 3 [19] |
| | | | | 3.53 [20] |
| | TNFSF12 (*TWEAK*) | Tumor necrosis factor ligand superfamily member 12 | ND | <1 [19] |
| | *NFKBIA* | Nuclear factor of kappa light polypeptide gene enhancer in B-cells inhibitor alpha | ND | 1.41 [20] |
| **DNA-repair pathway**<br>Nucleotide excision repair, interstrand cross-links repair and double-strand breaks repair efficiency correlates with drug resistance development and therapeutic outcome | *TP53* | Tumor protein P53 | Significant negative [25]. | 8 [4] |
| | | | | 15 [17] |
| | | | | 8 [3] |
| | | | | 3 [25] |
| | | | | 5 [19] |
| | | | | 5.66 [20] |
| | *ATM* | Ataxia telangiectasia mutated serine/threonine kinase | Impaired [25]. | 3 [25] |
| | | | | 3 [19] |
| | | | | 4.32 [20] |
| | *ATR* | Ataxia telangiectasia and Rad3-related protein serine/threonine kinase | Impaired [25]. | 1,3 [25] |
| | | | | 1 [19] |
| | *BRCA2* | Breast cancer type 2 susceptibility protein | ND | 0.43 [25] |
| | *BRCA1* | Breast cancer type 1 susceptibility protein | ND | 0.22 [25] |
| **RNA interactions**<br>Altered RNA metabolism may cause changes in levels of | *DIS3* | *DIS3* homolog, exosome endoribonuclease and 3'-5' exoribonuclease Associated with IGH translocations t(4;14), t(11;14) and t(14;16)and deletions of 13q14 [28]. | ND | 11 [4] |
| | | | | 11 [3] |
| | | | | 11 [28] |
| | | | | 8.6 [25] |

| | | | | |
|---|---|---|---|---|
| oncogenes, tumor suppressor or DNA repair genes | | | | 10 [19] |
| | | | | 9.98 [20] |
| | *FAM46C* | Family with sequence similarity 46, member C. | Worse [29]. Augment MM cell growth and survival [30]. | 13 [4] |
| | | | | 11 [31] |
| | | | | 11 [3] |
| | | | | 5.6 [25] |
| | | | | 9 [19] |
| | | | | 9.35 [20] |
| | *RPL10* | 60S ribosomal protein L10 | ND | 2 [19] |
| | *RPL5* | 60S ribosomal protein L5 | Better response to proteasome inhibitors, such as bortezomib [32] | ≥20 deletions [32] |
| | *SF3B1* | Splicing factor 3b subunit | ND | 1.73 [20] |
| | *RPS3A* | Ribosomal protein S3A | ND | 1 [19] |
| | *FCF1* | rRNA-processing protein FCF1 homolog | ND | <1 [19] |
| | *PABPC1* | Poly(A) binding protein cytoplasmic 1 | ND | 4 [19] |
| **Protein homeostasis** Response to unfolded/misfolded protein is important for efficient production of IG and normal functioning of plasma cells. | *LRRK2* | Leucine-rich repeat serine/threonine-protein kinase 2 LRKK2 phosphorylates translation initiation factor 4E-binding protein (4EBP) | ND | 8 [4] |
| | *PNRC1* | Proline rich nuclear receptor coactivator 1 | ND | 5.3 [4] |
| | *ALOX12B* | Arachidonate 12-lipoxygenase, 12R type | ND | 8 [4] |
| | *HLA-A* | Major histocompatibility complex, class I, A | ND | 5.3 [4] |
| | *MAGED1* | Melanoma-associated antigen family member D1 | ND | 5.3 [4] |
| | *PSMB5* | Proteasome 20S Subunit Beta 5 Mutations may confer resistance to proteasome inhibitors [33] | ND | 1.5 [33] |
| | *PSMB8* | Proteasome 20S Subunit Beta 8 Mutations may confer resistance to proteasome inhibitors [33] | ND | <1 [33] |
| | *PSMB9* | Proteasome 20S Subunit Beta 9 Mutations may confer resistance to proteasome inhibitors [33] | ND | <1 [33] |
| | *PSMD1* | Proteasome 26S Subunit, Non-ATPase 1 Mutations may confer resistance to proteasome inhibitors [33] | ND | 2 [33] |
| | *PSMG2* | Proteasome Assembly Chaperone 2 Mutations may confer resistance to proteasome inhibitors [33] | ND | <1 [33] |
| **Proteins forming fibrin clot** | *F3* | Coagulation factor III, tissue factor | ND | 2.6 [4] |
| | *F5* | Coagulation Factor V | ND | 2.6 [4] |
| | *FGA* | Fibrinogen alpha chain | ND | 5.3 [4] |

| | | | | |
|---|---|---|---|---|
| | *FGG* | Fibrinogen gamma chain | ND | 2.6 [4] |
| | *TFPI* | Tissue factor pathway inhibitor | ND | 2.6 [4] |
| **Epigenetics, chromatin remodeling, histones and histone modifying enzymes** Chromatin state is an important mechanism of regulation of gene expression and DNA damage response | *MLL (KMT2A)* | Myeloid/lymphoid or mixed-lineage leukemia 1 Lysine Methyltransferase 2A | ND | 2.6 [4] |
| | | | | 1.7 [25], [34] |
| | *MLL3 (KMT2C)* | Myeloid/lymphoid or mixed-lineage leukemia 3 lysine methyltransferase 2C | ND | 2.6 [4] |
| | | | | 3.14 [20] |
| | | | | 1.5 [34] |
| | *MLL2 (KMT2B)* | Myeloid/lymphoid or mixed-lineage leukemia 2 Lysine methyltransferase 2D | ND | 5.3 [4] |
| | *MLL5 (KMT2E)* | Myeloid/lymphoid or mixed-lineage leukemia 5 Lysine methyltransferase 2E | ND | 1.08 [34] |
| | *KDM6A (UTX)* | Lysine (K)-specific demethylase 6A | ND | 1.89 [20] |
| | *NSD2 (WHSC1)* | Nuclear receptor binding SET domain protein 2 | ND | 5.3 [4] |
| | | | | 2.6 indel [4] |
| | | | | 5.3 translocation [4] |
| | *NSD3 (WHSC1L1)* | Nuclear receptor binding SET domain protein 3 | ND | 5.3 [4] |
| | *ZFHX4* | Zinc finger homeobox protein 4 | ND | 4 [25] |
| | *CHD4* | Chromodomain helicase DNA binding protein 4 | ND | 2 [25] |
| | *ATRX* | Alpha thalassemia/mental retardation syndrome X-linked chromatin remodeler | ND | 2.51 [20] |
| | *EP300* | Histone lysine acetyltransferase p300, associated with t(14;16) | ND | 2.36 [20] |
| | *CREBBP* | Histone lysine acetyltransferase | ND | 0.65-3.85 [34] |
| | | | | 2.4 [20] |
| | *DNMT3A* | DNA (cytosine-5)-methyltransferase | ND | 0.43 [25] |
| | | | | 1.89 [20] |
| | *TET2* | Tet methylcytosine dioxygenase 2 | ND | 2.12 [20] |
| | *KDM5C* | Lysine demethylase 5 | ND | 2.12 [20] |
| | *ARID2* | AT-rich interaction domain | ND | 1.89 [20] |
| | *NCOR1* | Nuclear Receptor Corepressor 1 | ND | 1.49 [20] |
| | *SETD2* | SET Domain Containing Histone Lysine Methyltransferase 2 | ND | 2.36 [20] |
| | *SETDB1* | SET Domain Bifurcated Histone Lysine Methyltransferase 1 | ND | 1 [34] |
| | *EHMT2* | Euchromatic Histone Lysine Methyltransferase 2 | ND | 1 [34] |
| | *HIST1H1B* | Histone cluster 1 H1 family member B | ND | 1.4 [13] |
| | *HIST1H1D* | Histone cluster 1 H1 family member D | ND | 1 [13] |
| | *HIST1H1C* | Histone cluster 1 H1 family member C | ND | 3 [17] |

| | | | | |
|---|---|---|---|---|
| | | | | 2.59 [34] |
| | *HIST1H1E* | Histone cluster 1 H1 family member E | ND | 2.8 [25] |
| | | | | 4 [19] |
| | | | | 4,08 [20] |
| | | | | 4.7 [13] |
| | *HIST1H2BK* | Histone cluster 1 H2B family member K | ND | 1 [13] |
| | *HIST1H4H* | Histone cluster 1 H4 family member H | ND | <1 [19] |
| **Cell cycle pathway** Mutations in the cell cycle pathway lead to unregulated proliferation of plasma cells | *CCND1* | Cyclin D1 A regulator of CDK kinases MGUS, MM Significantly mutated in the t(11;14) subgroup [25]. May result from somatic hypermutation driven by the AID [17] | Negative [25]. | 5.3 [4] |
| | | | | 2.1 [25] |
| | | | | 2 [19] |
| | *RB1* | Retinoblastoma-associated protein, transcriptional corepressor 1 A negative regulator of the cell cycle | ND Bi-allelic inactivation portends poor prognosis | 3 [3] |
| | | | | 1.5 [25] |
| | | | | 2 [19] |
| | | | | 1.96 [20] |
| | *CDKN1B* | Cyclin dependent kinase inhibitor 1B | ND | 1.1 [20] |
| | *CDKN2C* | Cyclin Dependent Kinase Inhibitor 2C | Worse overall survival in MM patients who received ASCT [29]. | 9 deletion [20] |
| | | | | 33 [35] |
| **Transcriptional regulation (DNA interactions) and MYC interactions** Altered transcription causes oncogene activation, activation of plasma cell proliferation and apoptosis disregulation | *MAX* | MYC Associated Factor X. Transcription factor | ND | 2.4 [25] |
| | | | | 3 [19] |
| | | | | 3.14 [20] |
| | *FUBP1* | Far upstream element binding protein 1 | ND | 1.34 [20] |
| | *EGR1* | Early growth response protein 1 Significantly mutated in the hyperdiploid samples [25]. IMiD target genes. Associated with hypermutation as a consequence of AID activity [3] | Positive [25]. | 4 [3] |
| | | | | 3.5 [25] |
| | | | | 4 [19] |
| | | | | 4.79 [20] |
| | *IRF4* | Interferon regulatory factor 4. Association with t(11;14) [25]. IMiD target gene. Transcriptional regulator of PRDM1. CD8(+) dendritic cell differentiation | Positive [25]. | 5.3 [4] |
| | | | | 5 [36] |
| | | | | 3.2 [25] |
| | | | | 3 [19] |
| | | | | 3.14 [20] |
| | *PRDM1* | Beta-interferon gene positive regulatory domain I-binding factor. Plasmacytic differentiation. Promote survival of transformed plasma cells [37] | ND | 5.3 [4] |
| | | | | 5 [3] |
| | | | | 2 [19] |

| | | | | |
|---|---|---|---|---|
| | | | | 1.81 [20] |
| | *ZNF208* | Zinc finger protein 208 | ND | 3 [19] |
| | *HIF1A* | Hypoxia-inducible factor 1-alpha the adaptive response to hypoxia | ND | 0.22 [25] |
| | *RARA* | Retinoic acid receptor alpha regulation of development, differentiation, apoptosis, granulopoeisis, and transcription of clock genes | ND | 0.22 [25] |
| | *MAF* | V-Maf avian musculoaponeurotic fibrosarcoma oncogene homolog embryonic lens fiber cell development, increased T-cell susceptibility to apoptosis, chondrocyte terminal differentiation | ND | 1.65 [20] |
| | *IKZF1* | IKAROS family zinc finger protein 1, DNA-binding protein Ikaros, DNA-binding transcription regulator of hematopoietic cell differentiation | ND | 2 [38] |
| | | | | <1% of cases [17] |
| | *IKZF3* | IKAROS family zinc finger protein 3, DNA-binding protein Aiolos, transcription regulator of hematopoietic cell differentiation | ND | <1% of cases [17] |
| | *CRBN* | Cereblon, component of the cullin ring E3 ubiquitin ligase complex | Negative [38,39] | 12 [38] |
| | *CUL4A* | Component of cullin-RING-based E3 ubiquitin-protein ligase complex | ND | 6 [38] |
| | | | | <1% of cases [17] |
| | *MAFB* | V-Maf avian musculoaponeurotic fibrosarcoma oncogene homolog B the regulation of lineage-specific hematopoiesis | ND | 0.79 [20] |
| **Adhesion and motility** | *ABL1* | Tyrosine-protein kinase cytoskeleton remodeling DNA-damage response and apoptosis | ND | 0.43 [25] |
| | *ACTG1* | Actin Gamma 1 | ND | 2 [3] |
| | | | | 3 [19] |
| | | | | 3.22 [20] |
| | *DNAH5* | Dynein heavy chain 5 | ND | 5 [19] |
| | *MYH2* | Myosin-2 | ND | 3 [19] |
| | *NCKAP5* | Nck-associated protein 5 | Adverse [25]. | 2.2 [25] |
| | *FAM154B* | Family with sequence similarity 154, member B Stabilizer of axonemal microtubules 2 | ND | <1 [19] |
| **Others or multiple functions** | *IGLL5* | Immunoglobulin Lambda Like Polypeptide 5 | High risk of relapse and progressive disease [40] | 16 [40] |
| | *KLHL6* | Kelch-like family member 6, predicted oncogene, involved in B-cell receptor (BCR) signaling | ND | 2.8 [20] |
| | *BMP2K* | BMP-2-inducible protein kinase. Skeletal development | ND | 2 [19] |
| | *XBP1* | X-box-binding protein 1 | ND | 5 [4] |

| | | | | |
|---|---|---|---|---|
| | | Transcription factor involved in the unfolded protein response. Differentiation of B lymphocytes to plasma cells and production of immunoglobulins. The IRE1$\alpha$/XBP1 pathway is involved in both c-Myc- and N-Myc-driven cancers [41]. | | |
| | *SGPP1* | Sphingosine-1-phosphate phosphohydrolase 1. Epidermis development keratinocyte differentiation | ND | 1 [19] |
| | *LEMD2* | Lamina-associated polypeptide-emerin-MAN1 domain containing 2. Nuclear structure organization myoblast differentiation | ND | <1 [19] |
| | *FBXO4* | F-box only protein 4, phosphorylation-dependent ubiquitination | ND | 1 [19] |
| | *FTL* | Ferritin light chain, iron homeostasis | ND | <1 [19] |
| | *IDH1* | Isocitrate dehydrogenase (NADP(+)) 1, cytosolic metabolism and energy production | ND | 0.55 [20] |
| | *IDH2* | Isocitrate dehydrogenase (NADP(+)) 2, mitochondria, metabolism and energy production | ND | 0.22 [25]<br>0.39 [20] |
| | *PTH2* | Parathyroid hormone 2, neuropeptide | ND | 1 [19] |
| | *BAX* | BCL2 associated X, apoptosis regulator | ND | <1 [19] |
| | *C8orf86* | Chromosome 8 open reading frame 86, RNA gene | ND | <1 [19] |
| | *CELA1* | Chymotrypsin like elastase 1 | ND | <1 [19] |
| | *OR5M1* | Olfactory receptor family 5 subfamily M member 1, the perception of a smell | ND | 1 [19] |
| | *OR9G1* | Olfactory receptor family 9 subfamily G member 1 the perception of a smell | ND | <1 [19] |
| | *KIF5B* | Kinesin-1 heavy chain centrosome and nuclear positioning during mitotic entry | ND | 0.22 [25] |
| | *MPL* | Myeloproliferative leukemia protein, proto-oncogene, thrombopoietin receptor, regulator of megakaryopoiesis and platelet production | ND | 0.22 [25] |
| | *PIK3CA* | Phosphatidylinositol 4,5-bisphosphate 3-kinase catalytic subunit alpha isoform, endothelial cell migration angiogenesis glucose metabolic process | ND | 0.22 [25] |
| | *SAMHD1* | SAM and HD domain containing deoxynucleoside triphosphate triphosphohydrolase 1crbn, innate immune response | ND | 2 [19] |
| | *HUWE1* | HECT, UBA and WWE domain containing E3 ubiquitin protein ligase 1, ubiquitination of the anti-apoptotic protein Mcl1 and the p53 tumor suppressor | ND | 5.73 [20] |
| | *UBR5* | Ubiquitin protein ligase E3 component N-recognin 5 | ND | 3.85 [20] |

| | | | | |
|---|---|---|---|---|
| | *SP140* | SP140 nuclear body protein, repressor of genes implicated in inflammation | ND | 3 [19]<br>2.59 [20] |
| | *TBC1D29* | Putative TBC1 domain family member 29 | ND | 2 [19] |
| | *TGDS* | TDP-Glucose 4,6-Dehydratase, metabolism | ND | 2 [19]<br>1.26 [20] |
| | *RPN1* | Ribophorin I, Subunit of the oligosaccharyl transferase complex Part of the regulatory subunit of the 26S proteasome | ND | <1 [19] |
| **Mitochondrial**<br>Mutations have impact in sugar metabolism, apoptosis, cell cycle, cell growth, signaling and drug response | *MT-ND5* | Mitochondrially encoded NADH:Ubiquinone oxidoreductase core subunit 5 | ND | 29 [42] |
| | *MT-ND4* | Mitochondrially encoded NADH:Ubiquinone oxidoreductase core subunit 4 | ND | 24 [42] |
| | *MT-CO1* | Mitochondrially encoded cytochrome C oxidase I | ND | 20 [42] |
| | *MT-ND1* | Mitochondrially encoded NADH:Ubiquinone oxidoreductase core subunit 1 | ND | 15 [42] |
| | *MT-CYB* | Mitochondrially encoded cytochrome B | ND | 14 [42] |
| | *MT-ND2* | Mitochondrially encoded NADH:Ubiquinone oxidoreductase core subunit 2 | ND | 11 [42] |
| | *MT-CO3* | Mitochondrially encoded cytochrome C oxidase III | ND | 9 [42] |
| | *MT-ATP6* | Mitochondrially encoded ATP synthase membrane subunit 6 | ND | 6 [42] |
| | *MT-CO2* | Mitochondrially encoded cytochrome C oxidase II | ND | 6 [42] |
| | *MT-ND6* | Mitochondrially encoded NADH:Ubiquinone oxidoreductase core subunit 6 | ND | 5 [42] |
| | *MT-ATP8* | Mitochondrially encoded ATP synthase membrane subunit 8 | ND | 3 [42] |
| | *MT-ND3* | Mitochondrially encoded NADH:Ubiquinone oxidoreductase core subunit 3 | ND | 3 [42] |

ND – not determined, or impact is hard to estimate.

## References


1. Liu, R.; Gao, Q.; Foltz, S.M.; Fowles, J.S.; Yao, L.; Wang, J.T.; Cao, S.; Sun, H.; Wendl, M.C.; Sethuraman, S.; et al. Co-evolution of tumor and immune cells during progression of multiple myeloma. *Nature Communications* **2021**, *12*, 2559, doi:10.1038/s41467-021-22804-x.
2. Rustad, E.H.; Yellapantula, V.; Leongamornlert, D.; Bolli, N.; Ledergor, G.; Nadeu, F.; Angelopoulos, N.; Dawson, K.J.; Mitchell, T.J.; Osborne, R.J.; et al. Timing the initiation of multiple myeloma. *Nat Commun* **2020**, *11*, 1917, doi:10.1038/s41467-020-15740-9.
3. Lohr, J.G.; Stojanov, P.; Carter, S.L.; Cruz-Gordillo, P.; Lawrence, M.S.; Auclair, D.; Sougnez, C.; Knoechel, B.; Gould, J.; Saksena, G.; et al. Widespread genetic heterogeneity in multiple myeloma: implications for targeted therapy. *Cancer Cell* **2014**, *25*, 91-101, doi:10.1016/j.ccr.2013.12.015.
4. Chapman, M.A.; Lawrence, M.S.; Keats, J.J.; Cibulskis, K.; Sougnez, C.; Schinzel, A.C.; Harview, C.L.; Brunet, J.P.; Ahmann, G.J.; Adli, M.; et al. Initial genome sequencing and analysis of multiple myeloma. *Nature* **2011**, *471*, 467-472, doi:10.1038/nature09837.

5. Bolli, N.; Maura, F.; Minvielle, S.; Gloznik, D.; Szalat, R.; Fullam, A.; Martincorena, I.; Dawson, K.J.; Samur, M.K.; Zamora, J.; et al. Genomic patterns of progression in smoldering multiple myeloma. *Nat Commun* **2018**, *9*, 3363, doi:10.1038/s41467-018-05058-y.
6. Oben, B.; Froyen, G.; Maclachlan, K.H.; Leongamornlert, D.; Abascal, F.; Zheng-Lin, B.; Yellapantula, V.; Derkach, A.; Geerdens, E.; Diamond, B.T.; et al. Whole-genome sequencing reveals progressive versus stable myeloma precursor conditions as two distinct entities. *Nat Commun* **2021**, *12*, 1861, doi:10.1038/s41467-021-22140-0.
7. Hollein, A.; Twardziok, S.O.; Walter, W.; Hutter, S.; Baer, C.; Hernandez-Sanchez, J.M.; Meggendorfer, M.; Haferlach, T.; Kern, W.; Haferlach, C. The combination of WGS and RNA-Seq is superior to conventional diagnostic tests in multiple myeloma: Ready for prime time? *Cancer Genet* **2020**, *242*, 15-24, doi:10.1016/j.cancergen.2020.01.001.
8. Bustoros, M.; Sklavenitis-Pistofidis, R.; Park, J.; Redd, R.; Zhitomirsky, B.; Dunford, A.J.; Salem, K.; Tai, Y.T.; Anand, S.; Mouhieddine, T.H.; et al. Genomic Profiling of Smoldering Multiple Myeloma Identifies Patients at a High Risk of Disease Progression. *J Clin Oncol* **2020**, JCO2000437, doi:10.1200/JCO.20.00437.
9. Manzoni, M.; Marchica, V.; Storti, P.; Ziccheddu, B.; Sammarelli, G.; Todaro, G.; Pelizzoni, F.; Salerio, S.; Notarfranchi, L.; Pompa, A.; et al. Application of Next-Generation Sequencing for the Genomic Characterization of Patients with Smoldering Myeloma. *Cancers (Basel)* **2020**, *12*, doi:10.3390/cancers12051332.
10. Landau, H.J.; Yellapantula, V.; Diamond, B.T.; Rustad, E.H.; Maclachlan, K.H.; Gundem, G.; Medina-Martinez, J.; Ossa, J.A.; Levine, M.F.; Zhou, Y.; et al. Accelerated single cell seeding in relapsed multiple myeloma. *Nature Communications* **2020**, *11*, 3617, doi:10.1038/s41467-020-17459-z.
11. Misund, K.; Keane, N.; Stein, C.K.; Asmann, Y.W.; Day, G.; Welsh, S.; Van Wier, S.A.; Riggs, D.L.; Ahmann, G.; Chesi, M.; et al. MYC dysregulation in the progression of multiple myeloma. *Leukemia* **2020**, *34*, 322-326, doi:10.1038/s41375-019-0543-4.
12. Buedts, L.; Smits, S.; Ameye, G.; Lehnert, S.; Ding, J.; Delforge, M.; Vermeesch, J.; Boeckx, N.; Tousseyn, T.; Michaux, L.; et al. Ultra-low depth sequencing of plasma cell DNA for the detection of copy number aberrations in multiple myeloma. *Genes Chromosomes Cancer* **2020**, *59*, 465-471, doi:10.1002/gcc.22848.
13. Maura, F.; Bolli, N.; Angelopoulos, N.; Dawson, K.J.; Leongamornlert, D.; Martincorena, I.; Mitchell, T.J.; Fullam, A.; Gonzalez, S.; Szalat, R.; et al. Genomic landscape and chronological reconstruction of driver events in multiple myeloma. *Nat Commun* **2019**, *10*, 3835, doi:10.1038/s41467-019-11680-1.
14. Hoang, P.H.; Cornish, A.J.; Dobbins, S.E.; Kaiser, M.; Houlston, R.S. Mutational processes contributing to the development of multiple myeloma. *Blood Cancer J* **2019**, *9*, 60, doi:10.1038/s41408-019-0221-9.
15. Barwick, B.G.; Neri, P.; Bahlis, N.J.; Nooka, A.K.; Dhodapkar, M.V.; Jaye, D.L.; Hofmeister, C.C.; Kaufman, J.L.; Gupta, V.A.; Auclair, D.; et al. Multiple myeloma immunoglobulin lambda translocations portend poor prognosis. *Nat Commun* **2019**, *10*, 1911, doi:10.1038/s41467-019-09555-6.
16. Boyle, E.M.; Davies, F.E.; Deshpande, S.; Tytarenko, R.G.; Ashby, C.; Wang, Y.; Wardell, C.P.; Bauer, M.A.; Johnson, S.K.; Schinke, C.D.; et al. Analysis of the Sub-Clonal Structure of Smoldering Myeloma over Time Provides a New Means of Disease Monitoring and Highlights Evolutionary Trajectories Leading to Myeloma. *Blood* **2019**, *134*, 4333-4333, doi:10.1182/blood-2019-126679.
17. Bolli, N.; Avet-Loiseau, H.; Wedge, D.C.; Van Loo, P.; Alexandrov, L.B.; Martincorena, I.; Dawson, K.J.; Iorio, F.; Nik-Zainal, S.; Bignell, G.R.; et al. Heterogeneity of genomic evolution and mutational profiles in multiple myeloma. *Nat Commun* **2014**, *5*, 2997, doi:10.1038/ncomms3997.
18. Maura, F.; Petljak, M.; Lionetti, M.; Cifola, I.; Liang, W.; Pinatel, E.; Alexandrov, L.B.; Fullam, A.; Martincorena, I.; Dawson, K.J.; et al. Biological and prognostic impact of APOBEC-induced mutations in the spectrum of plasma cell dyscrasias and multiple myeloma cell lines. *Leukemia* **2018**, *32*, 1044-1048, doi:10.1038/leu.2017.345.
19. Hoang, P.H.; Dobbins, S.E.; Cornish, A.J.; Chubb, D.; Law, P.J.; Kaiser, M.; Houlston, R.S. Whole-genome sequencing of multiple myeloma reveals oncogenic pathways are targeted somatically through multiple mechanisms. *Leukemia* **2018**, *32*, 2459-2470, doi:10.1038/s41375-018-0103-3.
20. Walker, B.A.; Mavrommatis, K.; Wardell, C.P.; Ashby, T.C.; Bauer, M.; Davies, F.E.; Rosenthal, A.; Wang, H.; Qu, P.; Hoering, A.; et al. Identification of novel mutational drivers reveals oncogene dependencies in multiple myeloma. *Blood* **2018**, *132*, 587-597, doi:10.1182/blood-2018-03-840132.
21. Lagana, A.; Beno, I.; Melnekoff, D.; Leshchenko, V.; Madduri, D.; Ramdas, D.; Sanchez, L.; Niglio, S.; Perumal, D.; Kidd, B.A.; et al. Precision Medicine for Relapsed Multiple Myeloma on the Basis of an Integrative Multiomics Approach. *JCO Precis Oncol* **2018**, *2018*, doi:10.1200/PO.18.00019.
22. Manojlovic, Z.; Christofferson, A.; Liang, W.S.; Aldrich, J.; Washington, M.; Wong, S.; Rohrer, D.; Jewell, S.; Kittles, R.A.; Derome, M.; et al. Comprehensive molecular profiling of 718 Multiple Myelomas reveals significant differences in mutation frequencies between African and European descent cases. *PLoS Genet* **2017**, *13*, e1007087, doi:10.1371/journal.pgen.1007087.
23. Bustoros, M.; Park, J.; Salem, K.Z.; Liu, C.-J.; Capelletti, M.; Huynh, D.; Tai, Y.-T.; Mouhieddine, T.H.; Freeman, S.; Ha, G.; et al. Next Generation Sequencing Identifies Smoldering Multiple Myeloma Patients with a High Risk of Disease Progression. *Blood* **2017**, *130*, 392-392, doi:10.1182/blood.V130.Suppl_1.392.392.

24. Maura, F.; Bolli, N.; Minvielle, S.; Gloznik, D.; Szalat, R.; Fullam, A.; Martincorena, I.; Samur, M.K.; Tarpey, P.; Davies, H.; et al. Analysis of Mutational Signatures Suggest That Aid Has an Early and Driver Role in Multiple Myeloma. *Blood* **2016**, *128*, 116-116, doi:10.1182/blood.V128.22.116.116.
25. Walker, B.A.; Boyle, E.M.; Wardell, C.P.; Murison, A.; Begum, D.B.; Dahir, N.M.; Proszek, P.Z.; Johnson, D.C.; Kaiser, M.F.; Melchor, L.; et al. Mutational Spectrum, Copy Number Changes, and Outcome: Results of a Sequencing Study of Patients With Newly Diagnosed Myeloma. *J Clin Oncol* **2015**, *33*, 3911-3920, doi:10.1200/JCO.2014.59.1503.
26. Zhao, S.; Choi, M.; Heuck, C.; Mane, S.; Barlogie, B.; Lifton, R.P.; Dhodapkar, M.V. Serial exome analysis of disease progression in premalignant gammopathies. *Leukemia* **2014**, *28*, 1548-1552, doi:10.1038/leu.2014.59.
27. Bolli, N.; Biancon, G.; Moarii, M.; Gimondi, S.; Li, Y.; de Philippis, C.; Maura, F.; Sathiaseelan, V.; Tai, Y.T.; Mudie, L.; et al. Analysis of the genomic landscape of multiple myeloma highlights novel prognostic markers and disease subgroups. *Leukemia* **2018**, *32*, 2604-2616, doi:10.1038/s41375-018-0037-9.
28. Weißbach, S.; Langer, C.; Puppe, B.; Nedeva, T.; Bach, E.; Kull, M.; Bargou, R.; Einsele, H.; Rosenwald, A.; Knop, S.; et al. The molecular spectrum and clinical impact of DIS3 mutations in multiple myeloma. *British journal of haematology* **2015**, *169*, 57-70, doi:10.1111/bjh.13256.
29. Boyd, K.D.; Ross, F.M.; Walker, B.A.; Wardell, C.P.; Tapper, W.J.; Chiecchio, L.; Dagrada, G.; Konn, Z.J.; Gregory, W.M.; Jackson, G.H.; et al. Mapping of chromosome 1p deletions in myeloma identifies FAM46C at 1p12 and CDKN2C at 1p32.3 as being genes in regions associated with adverse survival. *Clin Cancer Res* **2011**, *17*, 7776-7784, doi:10.1158/1078-0432.CCR-11-1791.
30. Zhu, Y.X.; Shi, C.X.; Bruins, L.A.; Jedlowski, P.; Wang, X.; Kortum, K.M.; Luo, M.; Ahmann, J.M.; Braggio, E.; Stewart, A.K. Loss of FAM46C Promotes Cell Survival in Myeloma. *Cancer Res* **2017**, *77*, 4317-4327, doi:10.1158/0008-5472.CAN-16-3011.
31. Barbieri, M.; Manzoni, M.; Fabris, S.; Ciceri, G.; Todoerti, K.; Simeon, V.; Musto, P.; Cortelezzi, A.; Baldini, L.; Neri, A.; et al. Compendium of FAM46C gene mutations in plasma cell dyscrasias. *British journal of haematology* **2016**, *174*, 642-645, doi:10.1111/bjh.13793.
32. Hofman, I.J.F.; Patchett, S.; van Duin, M.; Geerdens, E.; Verbeeck, J.; Michaux, L.; Delforge, M.; Sonneveld, P.; Johnson, A.W.; De Keersmaecker, K. Low frequency mutations in ribosomal proteins RPL10 and RPL5 in multiple myeloma. *Haematologica* **2017**, *102*, e317-e320, doi:10.3324/haematol.2016.162198.
33. Barrio, S.; Stühmer, T.; Da-Viá, M.; Barrio-Garcia, C.; Lehners, N.; Besse, A.; Cuenca, I.; Garitano-Trojaola, A.; Fink, S.; Leich, E.; et al. Spectrum and functional validation of PSMB5 mutations in multiple myeloma. *Leukemia* **2019**, *33*, 447-456, doi:10.1038/s41375-018-0216-8.
34. Pawlyn, C.; Kaiser, M.F.; Heuck, C.; Melchor, L.; Wardell, C.P.; Murison, A.; Chavan, S.S.; Johnson, D.C.; Begum, D.B.; Dahir, N.M.; et al. The Spectrum and Clinical Impact of Epigenetic Modifier Mutations in Myeloma. *Clin Cancer Res* **2016**, *22*, 5783-5794, doi:10.1158/1078-0432.ccr-15-1790.
35. Tessoulin, B.; Moreau-Aubry, A.; Descamps, G.; Gomez-Bougie, P.; Maïga, S.; Gaignard, A.; Chiron, D.; Ménoret, E.; Le Gouill, S.; Moreau, P.; et al. Whole-exon sequencing of human myeloma cell lines shows mutations related to myeloma patients at relapse with major hits in the DNA regulation and repair pathways. *Journal of hematology & oncology* **2018**, *11*, 137, doi:10.1186/s13045-018-0679-0.
36. Ruiz-Heredia, Y.; Sánchez-Vega, B.; Onecha, E.; Barrio, S.; Alonso, R.; Martínez-Ávila, J.C.; Cuenca, I.; Agirre, X.; Braggio, E.; Hernández, M.T.; et al. Mutational screening of newly diagnosed multiple myeloma patients by deep targeted sequencing. *Haematologica* **2018**, *103*, e544-e548, doi:10.3324/haematol.2018.188839.
37. Lin, F.R.; Kuo, H.K.; Ying, H.Y.; Yang, F.H.; Lin, K.I. Induction of apoptosis in plasma cells by B lymphocyte-induced maturation protein-1 knockdown. *Cancer Res* **2007**, *67*, 11914-11923, doi:10.1158/0008-5472.can-07-1868.
38. Kortüm, K.M.; Mai, E.K.; Hanafiah, N.H.; Shi, C.X.; Zhu, Y.X.; Bruins, L.; Barrio, S.; Jedlowski, P.; Merz, M.; Xu, J.; et al. Targeted sequencing of refractory myeloma reveals a high incidence of mutations in CRBN and Ras pathway genes. *Blood* **2016**, *128*, 1226-1233, doi:10.1182/blood-2016-02-698092.
39. Gooding, S.; Ansari-Pour, N.; Towfic, F.; Ortiz Estévez, M.; Chamberlain, P.P.; Tsai, K.T.; Flynt, E.; Hirst, M.; Rozelle, D.; Dhiman, P.; et al. Multiple cereblon genetic changes are associated with acquired resistance to lenalidomide or pomalidomide in multiple myeloma. *Blood* **2021**, *137*, 232-237, doi:10.1182/blood.2020007081.
40. D'Agostino, M.; Innorcia, S.; Boccadoro, M.; Bringhen, S. Monoclonal Antibodies to Treat Multiple Myeloma: A Dream Come True. *Int J Mol Sci* **2020**, *21*, doi:10.3390/ijms21218192.
41. Chen, S.; Chen, J.; Hua, X.; Sun, Y.; Cui, R.; Sha, J.; Zhu, X. The emerging role of XBP1 in cancer. *Biomedicine & pharmacotherapy = Biomedecine & pharmacotherapie* **2020**, *127*, 110069, doi:10.1016/j.biopha.2020.110069.
42. Hoang, P.H.; Cornish, A.J.; Sherborne, A.L.; Chubb, D.; Kimber, S.; Jackson, G.; Morgan, G.J.; Cook, G.; Kinnersley, B.; Kaiser, M.; et al. An enhanced genetic model of relapsed IGH-translocated multiple myeloma evolutionary dynamics. *Blood Cancer Journal* **2020**, *10*, 101, doi:10.1038/s41408-020-00367-2.
